# *E. coli* do not count single molecules

**Published:** 2024-07-09

**Authors:** Henry H. Mattingly, Keita Kamino, Jude Ong, Rafaela Kottou, Thierry Emonet, Benjamin B. Machta

**Affiliations:** 1. Center for Computational Biology, Flatiron Institute; 2. Institute of Molecular Biology, Academia Sinica; 3. Molecular, Cellular, and Developmental Biology, Yale University; 4. Physics, Yale University; 5. QBio Institute, Yale University

## Abstract

Organisms must perform sensory-motor behaviors to survive. What bounds or constraints limit behavioral performance? Previously, we found that the gradient-climbing speed of a chemotaxing *Escherichia coli* is near a bound set by the limited information they acquire from their chemical environments (1). Here we ask what limits their sensory accuracy. Past theoretical analyses have shown that the stochasticity of single molecule arrivals sets a fundamental limit on the precision of chemical sensing (2). Although it has been argued that bacteria approach this limit, direct evidence is lacking. Here, using information theory and quantitative experiments, we find that *E. coli*’s chemosensing is *not* limited by the physics of particle counting. First, we derive the physical limit on the behaviorally-relevant information that any sensor can get about a changing chemical concentration, assuming that every molecule arriving at the sensor is recorded. Then, we derive and measure how much information *E. coli*’s signaling pathway encodes during chemotaxis. We find that *E. coli* encode two orders of magnitude less information than an ideal sensor limited only by shot noise in particle arrivals. These results strongly suggest that constraints other than particle arrival noise limit *E. coli*’s sensory fidelity.

## Introduction

Organisms must rapidly and accurately sense their environment, and then act on that sensory information to perform motor behaviors. Despite the importance of these processes for organisms’ survival, it is unclear what factors limit sensory fidelity and how this fidelity impacts behavioral performance (3). Past works have demonstrated that physics external to an organism often place fundamental limits on sensing accuracy and have argued that biological sensory systems might approach these limits (4,5,2,6–9). Alternatively, it is possible that other, system-specific constraints combined with demands on cellular resources are instead limiting (10–17). Understanding which constituent processes of a behavior limit performance would reveal relevant constraints on evolution and learning of sensory-motor behaviors.

*Escherichia coli* chemotaxis is an ideal system to study these questions. Bacteria use the chemotaxis system to navigate chemical gradients, which is important for fitness-relevant behaviors such as climbing quickly or localizing at sources (18–21). Furthermore, we understand in detail how *E. coli* sense and act on chemical signals (22–24). *E. coli* alternate between straight-swimming runs and randomly-reorienting tumbles (25). As they swim, the local concentrations of attractant chemicals change in time. These extracellular ligands bind to the cell’s transmembrane receptors, which modify the activity of receptor-associated CheA kinases inside the cell. CheA phosphorylates the diffusible response regulator CheY, which is dephosphorylated by CheZ. When conditions worsen, kinase activity increases, increasing CheYp concentration. CheYp then binds to the motor and increases the propensity to tumble, biasing the cell’s runs towards more favorable chemical environments.

We recently demonstrated that *E. coli* chemotaxis is information-limited: cells climb shallow gradients near a bound set by their sensory capabilities (1). First, we showed theoretically that the rate at which a cell encodes information about chemical signals sets an upper limit on its gradient-climbing speed. Then, through a combination of single-cell Förster resonance energy transfer (FRET) experiments and measurements of cells swimming in gradients, we found that a typical *E. coli* cell gets very little information—about 0.01 bits/s in a centimeter-long gradient—but efficiently uses this information to climb gradients at speeds near the theoretical limit. This suggests that a bacterium with a more accurate sensor would climb gradients faster, likely increasing their fitness.

What prevents *E. coli* from obtaining more information during chemotaxis? In their classic work, Berg and Purcell demonstrated that the stochastic arrival of particles at the cell surface places a fundamental limit on the accuracy of chemical sensing (2), regardless of its sensor’s molecular details. Since then, theoretical works have studied the effects of receptor binding (26–28), maximum-likelihood estimation (29), energy consumption with noisy readout molecules (10,30–32), time-varying concentrations (11,33,34), constant concentration ramps (8,35,36), and other factors (28,37) on this fundamental limit. Furthermore, several studies have argued that the sensitivity of bacteria’s chemosensing apparatus approaches the molecule-counting limit (2,8). However, it is still unclear whether this fundamental limit meaningfully constrains the information *E. coli* get about chemical signals, and thus their speed at climbing gradients. Answering this question has been challenging because it has been unclear how the fidelity of chemosensing relates to chemotaxis performance, and because of difficulties with measuring, quantifying, and interpreting cells’ internal encoding of external signals.

Here, we address these challenges with a combination of information theory and single-cell FRET measurements. Information theory allows us to quantify the fidelity of signal encoding in a cellular system, and single-cell FRET measurements give us a direct readout of the kinase activity in which *E. coli* encode environmental information. We first derive the physical limit on the rate at which an ideal sensor can acquire behaviorally-relevant information, set by ligand arrival noise. Next, we derive the rate at which *E. coli* encode this information in their kinase activity. By measuring signal statistics, kinase response functions, and fluctuations in kinase activity, we quantify both the physical limit and how much information a typical *E. coli* cell gets during chemotaxis. We find that *E. coli* get orders of magnitude less information than the physical limit. Therefore, when signals are weak and sensor quality matters, cells climb gradients much slower than an ideal, single-molecule-sensing agent could. Our work opens up new questions about what costs, constraints, or competing objectives prevent them from being closer to the physical limit.

### Chemotaxis requires information about the current time derivative of concentration

Determining whether particle arrival noise is a limiting factor during *E. coli* chemotaxis presents conceptual challenges. Cells process measurements of their chemical environment into internal states, like the activity of kinases and the concentrations of signaling molecules. However, the goal of the chemotaxis system is not to represent the current concentration with high accuracy per se, but instead to utilize the concentration signal to move up a chemical gradient. Thus, cells need to capture certain aspects of signals that are behaviorally-relevant, but not necessarily in a format which is simply interpretable to an observer. To quantify how accurate such internal representations are thus requires a mathematical understanding of what features of the concentration signal are relevant to chemotaxis.

**Figure 1:**
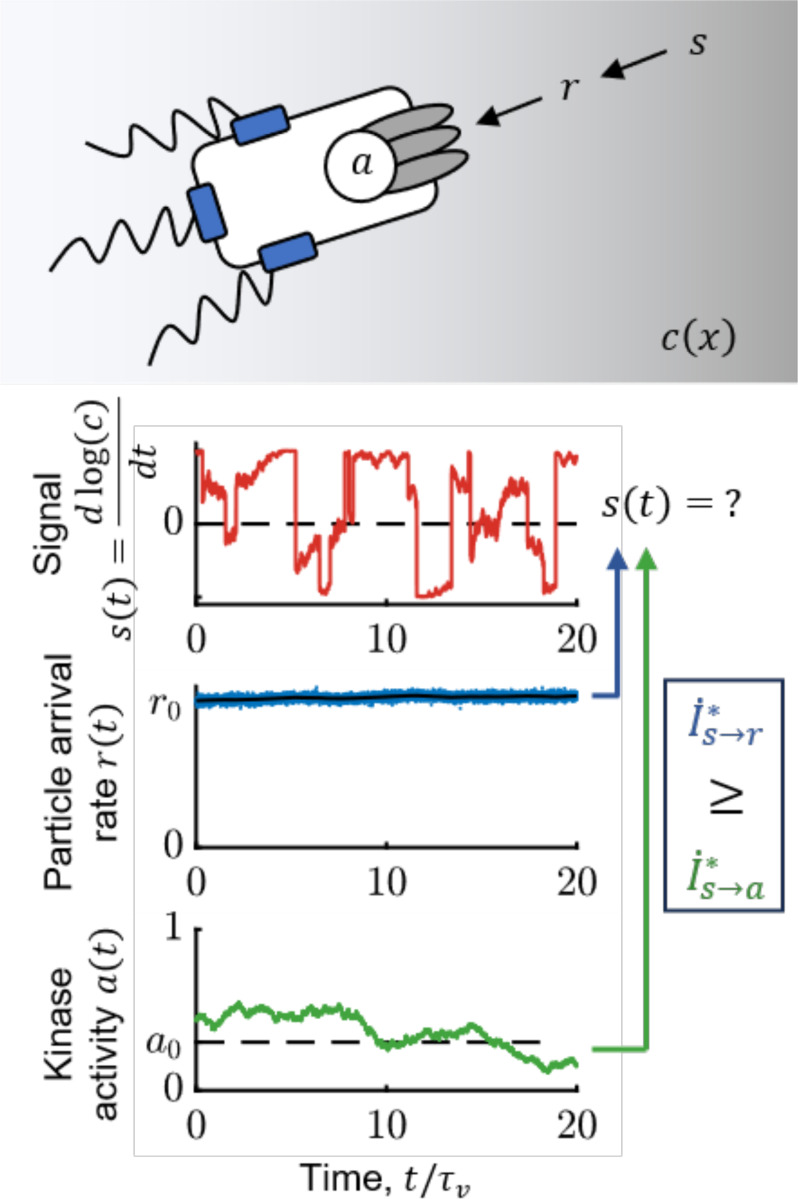
*E. coli* need to infer rate of change of attractant concentration from stochastic molecule arrivals. Top: Bacteria do not measure signal $s=\frac{d}{dt}\log(c)$ directly—instead, they can at best measure stochastic particle arrivals at rate r(t) at their transmembrane receptors. Receptor-associated kinases respond to ligand arrivals with changes in activity, a(t), and encode information about s(t), but also introduce additional noise. Bottom: Simulated traces of s(t) (red); r(t) (blue); $\langle r(t)\rangle =k_{D}\;c(t)$ (black); and kinase activity a(t) (green) for a cell exhibiting run-and-tumble motion in a shallow chemical gradient. $r_{0}$ is the background particle arrival rate, $r_{0}=k_{D}\;c_{0}$, and $a_{0}$ is the baseline level of kinase activity. The cell’s task is to infer s(t) from kinase activity a, and the fidelity of this inference is quantified by the transfer entropy rate, ${\dot{I}}_{s\to a}^{*}$. An ideal agent would directly estimate s(t) from the particle arrival rate r, without the noise in kinase activity, thus setting the physical limit, ${\dot{I}}_{s\to r}^{*}$. The simulation above was performed in a background concentration $c_{0}=1\;\text{μM}$ and gradient of steepness $g=0.3\;{\text{mm}}^{-1}$.

Our approach for addressing this builds on our recent work (1), where we identified the behaviorally-relevant information for *E. coli* chemotaxis. In particular, we showed that the amount of such information that the cell uses at the motor *determines* its gradient-climbing speed, $v_{d}\propto {\left(\dot{I}\right)}^{1/2}$. Furthermore, due to the data-processing inequality (38,39), the amount of this information in any intermediate variable *bounds* performance (see also SI). The key chemical signal that the cell needs to encode is the (relative) rate of change of concentration, $s(t)=\frac{d}{dt}\log(c)$ (Fig. 1). Then, the behaviorally-relevant information is the “transfer entropy rate” (40) from *current* signal, s(t), to a time-dependent variable x(t) that encodes the signal in its trajectory, {x}, up to time t:

(1) $${\dot{I}}_{s\to x}^{*}\equiv \underset{dt\to 0}{\lim}\frac{1}{dt}I(x(t+dt);s(t)|\{x\}),$$

where I(X;Y∣Z) is the mutual information between X and Y, conditioned on Z (38,41). Importantly, the current value of x(t) does not need to be an explicit representation of s(t); it just has to carry information about s(t) in its trajectory.

This points to a way of quantifying how molecule-counting noise limits behaviorally-relevant information for chemotaxis, and how *E. coli* compare to the limit. The stochastic arrival rate of ligand molecules at the cell surface, r(t), is the first quantity that a cell can physically measure that encodes information about signals s(t) (Fig. 1). Thus, the transfer entropy rate ${\dot{I}}_{s\to r}^{*}$ (i.e. with x=r in Eqn. 1) is a fundamental physical limit on the sensory information available for chemotaxis. An ideal agent would make navigation decisions based on a perfect readout of past particle arrivals {r}, but this process would still be noisy due to their inherent stochasticity. Then, *E. coli* encodes the signal in the activity of CheA kinases, {a}, from which downstream behavioral decisions are made. The data-processing inequality implies that ${\dot{I}}_{s\to r}^{*}\geq {\dot{I}}_{s\to a}^{*}$. Therefore, to compare *E. coli* to the physical limit, we must quantify the information about s(t) encoded in {a}, ${\dot{I}}_{s\to a}^{*}$ (i.e. with x=a in Eqn. 1). If ${\dot{I}}_{s\to a}^{*}$ is comparable to ${\dot{I}}_{s\to r}^{*}$, then *E. coli*’s signaling pathway acquires most of the information that is available in molecule arrivals. This comparison would allow us to determine whether *E. coli*’s chemotaxis performance is limited by the external physics of ligand diffusion or by other factors.

Our task now is to obtain closed form expressions for ${\dot{I}}_{s\to r}^{*}$ and ${\dot{I}}_{s\to a}^{*}$, and then quantify them with experimental measurements. In the SI (Eqn. 10), we show that this transfer entropy rate is equivalent to a *predictive* information rate (42–48),

(2) $${\dot{I}}_{s\to x}^{*}=-{\left[\partial_{\tau }I(s(t+\tau );\{x\})\right]}_{\tau =0}.$$

On the right, τ is a time interval into the future at which the signal s(t+τ) is predicted from past observations, {x}, making this a predictive information. Thus, the information about current signal s(t) that is *encoded* in past x is the same as the accuracy with which s(t) can be *estimated* from past x. We used this form to derive expressions for ${\dot{I}}_{s\to r}^{*}$ and ${\dot{I}}_{s\to a}^{*}$ (SI). Fig. 1 illustrates this problem, showing simulated time traces of signal s, particle arrival rate r, and kinase activity a. The goal of a chemotaxing *E. coli* is to construct an optimal running estimate of s(t). An ideal agent does this from observations {r}, whereas the cell only has access to past kinase activity {a}.

### Physical limit on information due to stochastic particle arrivals

We first derive an expression for the physical limit, ${\dot{I}}_{s\to r}^{*}$, from a model for the dynamics of s(t) and r(t). In static gradients, the signals a cell experiences are determined by their own motion in the gradient. Accordingly, in a gradient of steepness g=dlog(c)/dx, the signal is $s(t)=g\;v_{x}(t)$, where $v_{x}$ is the cell’s up-gradient velocity. As done previously (1,48), we consider a cell exhibiting run-and-tumble motion in a shallow gradient. In this regime, to leading order in g, the information rate only depends on the correlation function of up-gradient velocity in the absence of a gradient, V(t), since s is proportional to g. Thus, we approximate the signal as Gaussian, and its dynamics are fully characterized by the following correlation function:

(3) $$\langle s(t)\;s\left(t^{\prime }\right)\rangle =g^{2}V\left(t-t^{\prime }\right)=g^{2}\sigma_{v}^{2}\exp\left(-\frac{\left|t-t^{\prime }\right|}{\tau_{v}}\right).$$

Here, V(t) is the correlation function of $v_{x}$, $\sigma_{v}^{2}$ is the variance $v_{x}$, and $\tau_{v}$ is the signal correlation time, which depends on the cell’s mean run duration, the persistence of tumbles, and rotational diffusion (1,49).

We take particle arrival events to follow a Poisson process with time-varying rate $\langle r(t)\rangle =4\;D\;l\;c(t)=k_{D}\;c(t)$, where D is the diffusivity of the ligand and l is the diameter of a circular patch on the cell’s surface (2,28). If a sufficient number of particles arrive per run, $r_{0}\;\tau_{v}\gg 1$, which is valid in our experimental conditions, we can approximate the number of particles that arrive per unit time as Gaussian:

(4) $$r(t)=k_{D}\;c(t)+\sqrt{r_{0}}\;\xi (t).$$

Here, $r_{0}=k_{D}\;c_{0}$ is the background molecule arrival rate, where $c_{0}$ is the background concentration, and the noise is $\langle \xi (t)\xi \left(t^{\prime }\right)\rangle =\delta \left(t-t^{\prime }\right)$.

Next, since s(t) and {r} are each approximately Gaussian, the mutual information between them in Eqn. 2 has a known form (38) (SI Eqn. 13). In particular, it depends on $\sigma_{s|r}^{2}(\tau )$, the variance of the optimal estimate of s(t+τ) constructed from the past of r. Thus, the problem of deriving the physical limit reduces to solving $\sigma_{s|r}^{2}(\tau )$, which can be done using causal Wiener filtering theory (50–52) (see also (44,48,53–55)) (SI). In the SI, we derive the physical limit on behaviorally-relevant information for chemotaxis, which in the limit of shallow gradients reduces to:

(5) $${\dot{I}}_{s\to r}^{*}\approx \frac{1}{\tau_{v}}\frac{1}{4}\gamma_{r}.$$

Above, we have defined the dimensionless signal-to-noise ratio of particle arrivals, $\gamma_{r}=2\;r_{0}\;g^{2}\;\sigma_{v}^{2}\;\tau_{v}^{3}$. Eqn. 5 is valid when $\gamma_{r}\ll 1$, which defines the small-signal regime for ${\dot{I}}_{s\to r}^{*}$. We also provide a full expression for ${\dot{I}}_{s\to r}^{*}$ in the SI (Eqn. 44). The signal strength is proportional to $r_{0}^{2}$, while the noise is proportional to $r_{0}$. Thus, increasing the molecular arrival rate $r_{0}$, the gradient steepness g, or the variance of the up-gradient swimming speed $\sigma_{v}$ increases the signal-to-noise ratio of particle arrivals. Furthermore, the longer the cell maintains its heading, $\tau_{v}$, the more time it has to average out the noise of particle arrivals. Past work has shown that the relative error of estimating a constant time derivative scales as $1/T^{3}$, where T is the integration time (35). In chemotaxis, the longest reasonable integration time is the time scale on which the signal doesn’t change significantly, $\tau_{v}$. Therefore, a factor of $\tau_{v}^{3}$ appears in $\gamma_{r}$. The derivation of ${\dot{I}}_{s\to r}^{*}$ also provides the optimal kernel for constructing a running estimate of s(t) given past particle arrivals {r}, which we discuss in the SI (Fig. S4).

Above, we have modeled an ideal sensor that “absorbs” every molecule it senses (2). If the sensor cannot distinguish between new ligand arrival events and rebinding events, the bound is lower by an order-1 prefactor (28,37).

### Information encoded in *E. coli*’s CheA kinase activity

How do *E. coli* compare to the fundamental limit? To answer this, we need to derive and experimentally quantify the information, ${\dot{I}}_{s\to a}^{*}$, encoded in the activity a(t) of *E. coli*’s CheA kinases. This in turn requires models for both noise and responses of kinase activity.

As done before (1), in shallow gradients or for small signals, kinase activity can be described using linear response theory. In background particle arrival rate $r_{0}$ and with steady-state kinase activity $a_{0}$, then activity becomes:

(6) $$a(t)=a_{0}-\int_{-\infty }^{t}K_{r}\left(t-t^{\prime }\right)\left(r\left(t^{\prime }\right)-r_{0}\right)dt^{\prime }+\eta_{n}(t).$$

*E. coli* respond to a step increase in attractant concentration with a fast initial drop in kinase activity, followed by slow adaptation back to pre-stimulus levels (56). This response is captured by a phenomenological form for the response function:

(7) $$K_{r}(t)=G_{r}\left(\left(\frac{1}{\tau_{1}}+\frac{1}{\tau_{2}}\right)\exp\left(-\left(\frac{1}{\tau_{1}}+\frac{1}{\tau_{2}}\right)t\right)-\frac{1}{\tau_{2}}\exp\left(-\frac{t}{\tau_{2}}\right)\right)\text{Θ}(t),$$

where $G_{r}$ is the gain of the response to particle arrival rate r, $\tau_{1}$ is the fast initial response time, $\tau_{2}$ is the slow adaptation time, and Θ(t) is the Heaviside step function. This response function can equivalently be expressed in terms of responses to past signals s, with a related kernel K(t) that we used previously (1) $\left(K_{r}(t)=\frac{1}{r_{0}}\frac{d}{dt}K(t\right)$; Methods, Eqn. 15 below).

Noise in kinase activity is driven by a combination of stochastic particle arrivals and internally-driven fluctuations. Single-cell experiments have observed large, slow fluctuations in kinase activity on a time scale of 10 s (1,57–59). These are well-described as Gaussian, $\eta_{n}(t)$ in Eqn. 6, with correlation function:

(8) $$\langle \eta_{n}(t)\;\eta_{n}\left(t^{\prime }\right)\rangle =D_{n}\;\tau_{n}\;\exp\left(-\frac{\left|t-t^{\prime }\right|}{\tau_{n}}\right).$$

Here, $D_{n}$ is the diffusivity of slow noise in kinase activity, and $\tau_{n}$ is its correlation time. So far, it has not been possible to measure noise in kinase activity at time scales near or below $\tau_{1}$, but the noise cannot go below the level set by kinase responses to particle arrival noise. Thus, we construct a phenomenological noise model that agrees with experiments at low frequencies while obeying known physics at high frequencies. This consists of adding kinase responses to particle shot noise in Eqn. 4 to the slow fluctuations in Eqn. 8. Due to the adaptive nature of the signaling pathway, all the parameters that appear in the above Eqns. 7 and 8 can depend on the background particle arrival rate, $r_{0}$.

With this model, we can derive an expression for the information about signal encoded in kinase activity, ${\dot{I}}_{s\to a}^{*}$. As above, this reduces to deriving $\sigma_{s|a}^{2}(\tau )$, the variance of the signal s(t+τ) reconstructed from the past of kinase activity {a}, which can again be solved using Wiener filtering theory (SI). Furthermore, previous measurements (and measurements below) have shown that $\tau_{1}\ll \tau_{v}$ (1,60,61) and $\tau_{2}\approx \tau_{n}$ (1). Thus, in shallow gradients, we find that the information rate to kinase activity is:

(9) $${\dot{I}}_{s\to a}^{*}\approx \frac{1}{\tau_{v}}\frac{1}{4}\gamma_{a}\frac{\gamma_{r}/\gamma_{a}}{{\left(1+\sqrt{\gamma_{r}/\gamma_{a}}\right)}^{2}}.$$

Here, we have defined the dimensionless kinase signal-to-noise ratio $\gamma_{a}=\frac{G_{r}^{2}}{D_{n}}\;r_{0}^{2}\;g^{2}\;\sigma_{v}^{2}\;\tau_{v}$ and used $\gamma_{r}=2\;r_{0}\;g^{2}\;\sigma_{v}^{2}\;\tau_{v}^{3}$ from above. Eqn. 9 is valid when $\gamma_{a}\ll 1$, which defines the small-signal regime for ${\dot{I}}_{s\to a}^{*}$. We also provide a full expression for ${\dot{I}}_{s\to a}^{*}$ in the SI (Eqn. 89). An ideal cell with no internal noise sources would operate at the physical limit, Eqn. 5, corresponding to infinite signal-to-noise in kinase activity, $\gamma_{a}\to \infty$. Taking this limit in Eqn. 9 results in the expression for ${\dot{I}}_{s\to r}^{*}$ above (Eqn. 5). Conversely, a cell with internal noise would degrade information about the signal, and in the limit of large noise would have an information rate given by ${\dot{I}}_{s\to a}^{*}\approx \frac{1}{\tau_{v}}\frac{1}{4}\gamma_{a}$. The derivation of ${\dot{I}}_{s\to a}^{*}$ also provides the optimal kernel for constructing a running estimate of s(t) from past kinase activity {a}, which we discuss in the SI.

To compare the information *E. coli* get during chemotaxis to the physical limit, we must quantify ${\dot{I}}_{s\to a}^{*}$ and ${\dot{I}}_{s\to r}^{*}$ by measuring the parameters above from live cells.

### Single-cell measurements constrain signal and kinase properties

Next, we use single-cell tracking and FRET experiments to measure the parameters that characterize the signal statistics, kinase response function, and kinase noise statistics in multiple background concentrations of attractant. As the attractant, we used aspartate (Asp), to which the *E. coli* chemotaxis signaling pathway responds with the highest sensitivity among known attractants (62).

**Figure 2:**
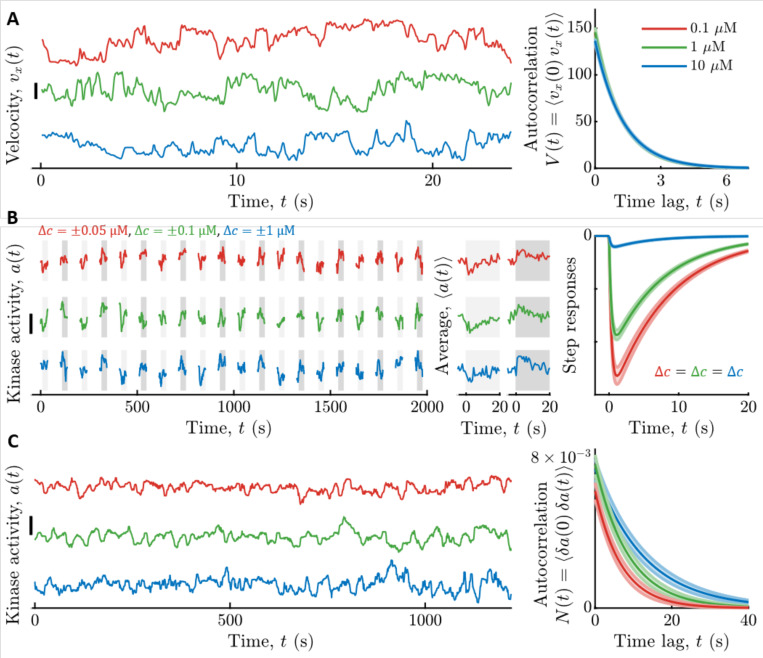
Measured signal statistics and kinase responses and fluctuations in different background ligand concentrations. **A)** Signal statistics. Left: Representative time series of up-gradient velocity $v_{x}$ from three individual cells are shown, one in each background concentration $c_{0}$. Scale bar is 20 μm/s. Cells were binned by the fraction of time they spend running, $P_{run}$, and the velocity autocorrelation function was computed by averaging over cells in the median bin $\left(P_{run}\approx 0.89\right)$. The parameters of the velocity autocorrelation function were then fit with a decaying exponential $V(t)=\sigma_{v}^{2}\exp\left(-\frac{t}{\tau_{v}}\right)$ to extract the velocity variance $\sigma_{v}^{2}$ and correlation time $\tau_{v}$. Right: Model fits for velocity autocorrelation functions are shown for each. $c_{0}$. The curves are on top of each other. Units on the y-axis are (μm/s)^2^. Throughout, line colors indicate $c_{0}$: Red: 0.1 μM Asp; Green: 1 μM Asp; Blue: 10 μM Asp, and shading is standard error of the mean (SEM). **B)** Linear responses. Left: Immobilized cells were continuously exposed with a constant background concentration $c_{0}$ of aspartate (Asp). The fraction of active kinases (kinase activity) was measured by FRET in blocks of 25 seconds, separated by 65 seconds without illumination. In each block, after 5 s, concentration was stepped up (light gray shading) or down (dark gray shading) around $c_{0}$, then maintained for 20 s, and then returned to $c_{0}$. Concentration step sizes Δc were different for each $c_{0}$ (shown above the panel). Shown are three representative cells, one from each $c_{0}$. Scale bar is 0.3. Middle: Average responses of the cells in the left panel to a step up (light gray) and step down (dark gray) of concentration. Single-cell responses were fit to the model in Eqn. 15 to extract single-cell parameters of the response function $K_{r}(t)$. Right: Using the median parameter values of the population, shown are model fits for kinase responses to a step increase in concentration of size Δc, for each background $c_{0}$. The gain of the response $G_{r}$ decreases with $c_{0}$. **C)** Noise statistics. Left: Fluctuations in kinase activity were measured in constant background concentrations. Representative time series from three cells are shown, one in each background concentration. Scale bar height is 0.3. Parameters of the slow noise autocorrelation function (Eqn. 8), were fit to single-cell traces using Bayesian filtering (SI). Right: Estimated noise autocorrelation functions for the median cell are shown, for each background concentration $c_{0}$. Units on the y-axis are kinase activity squared.

To quantify the parameters describing cell swimming statistics (Eqn. 3), and thus the signal statistics, $\sigma_{v}^{2}$ and $\tau_{v}$, we recorded trajectories of cells swimming in multiple uniform background concentrations of Asp: $c_{0}=0.1$, 1, and 10 μM (Fig. 2A). Single cells in the clonal population exhibited a range of swimming behaviors (57,63–69); thus, as before (1), we focus on cells with median values of the phenotypic parameters. We binned cells by the fraction of time they spent in the “run” state, $P_{run}$, and computed the velocity correlation function, V(t), among cells with the median $P_{run}$. The parameters $\sigma_{v}^{2}$ and $\tau_{v}$ in each background $c_{0}$ were then inferred by fitting the correlation functions with the decaying exponential in Eqn. 3. These parameters depended weakly on $c_{0}$, and their values in $c_{0}=1\;\text{μM}$ Asp were $\sigma_{v}^{2}=146\pm 5{\left(\text{μm}/s\right)}^{2}$ and $\tau_{v}=1.19\pm 0.01\;s$ (see Fig. S1AB for their values in all backgrounds).

We measured kinase response functions as before (1), using a microfluidic device in which we can deliver controlled chemical stimuli with high time resolution (~100 ms) (70). Cells immobilized in the device were delivered ten small positive and negative step changes of Asp concentration around multiple backgrounds $c_{0}$ (Fig. 2B; Methods). Kinase responses were measured in single cells through FRET (58,59,70–74) between CheZ-mYFP and CheY-mRFP. Then we fit each cell’s average response with the phenomenological response function $K_{r}(t)$ in Eqn. 7, and computed the population-median parameter values. However, $\tau_{1}=0$ estimated this way includes the dynamics of CheY-CheZ interactions, which are slower than the fast time scale of the kinases. We used $\tau_{1}=0$ for calculations below, which slightly overestimates the information rate ${\dot{I}}_{s\to a}^{*}$, making this a conservative choice in estimating where cells are relative to the bound. The adaptation time $\tau_{2}$ depended weakly on $c_{0}$ (in $c_{0}=1\;\text{μM}$, $\tau_{2}=7.4\pm 0.3\;s$) (Fig. S1D), but $G_{r}$ varied significantly with $c_{0}$: for $c_{0}=\{0.1,1,10\}\;\text{μM}$ we measured $G_{r}=\frac{1}{kD}\{3.2\pm 0.1,2.28\pm 0.05,0.251\pm 0.009\}$ (Fig. S1EF).

The dependence of $G_{r}$ on $c_{0}$ was consistent with the phenomenological Monod-Wyman-Changeux (MWC) model for kinase activity (23,75–77), which captures numerous experimental measurements (70,72–74,78). First, in the methods we note that $G_{r}=\frac{1}{r_{0}}G\left(c_{0}\right)$, where $G\left(c_{0}\right)$ is the MWC model gain (Eqn. 16 in the Methods below). The MWC model in turn predicts that $G\left(c_{0}\right)\approx G_{\infty }\frac{c_{0}}{c_{0}+K_{i}}$, where $K_{i}$ is the dissociation constant of two-state receptors for ligand when in their inactive state and $G_{\infty }$ is a constant (Methods). Thus, in low backgrounds where $c_{0}\ll K_{i}$ the cell is in the “linear-sensing” regime and $G_{r}=G_{\infty }\frac{1}{k_{D}K_{i}}$ is constant; in high backgrounds where $c_{0}⨠K_{i}$, cells transition to the “log-sensing” regime (79–81), with gain $G_{r}\approx G_{\infty }/r_{0}$. Fitting $G\left(c_{0}\right)$ to the MWC model, we estimated that $G_{\infty }=3.5\pm 0.1$ and $K_{i}=0.81\pm 0.04\;\text{μM}$.

Finally, we estimated the noise parameters of slow kinase fluctuations by measuring kinase activity in single cells experiencing constant Asp concentrations $c_{0}$ (Fig. 2C). The diffusivity $D_{n}$ and time scale $\tau_{n}$ of slow fluctuations in Eqn. 8 were extracted from these time series using Bayesian filtering (1,82) (Methods). We then computed the population-median parameter values. Both of these parameters depended weakly on $c_{0}$, and their values in $c_{0}=1\;\text{μM}$ were $D_{n}=8.1\pm 0.9\times 10^{-4}\;{\text{s}}^{-1}$ and $\tau_{n}=8.7\pm 0.9\;s$ (see Fig. S1CD for their values in all backgrounds).

### Comparing *E. coli* to the physical limit

We can now answer our central question: does the stochastic arrival of particles prevent *E. coli* from getting more information during chemotaxis? The remaining unknown needed to answer this is the diffusion-limited particle arrival rate constant, $k_{D}=4\;D\;l$. We take l=60nm (82) as a conservative lower estimate of the diameter of the receptor array and $D=800\;{\text{μm}}^{2}/\text{s}$ (83,84) as the ligand diffusivity. With these, we estimate that $k_{D}\approx 1.2\times 10^{5}\;{\text{s}}^{-1}\;{\text{μM}}^{-1}$, indicating that about 10^5^ independent molecules strike the cell’s receptor array per second in a background of $c_{0}=1\;\text{μM}$, which is comparable to previous estimates (2,8).

**Figure 3:**
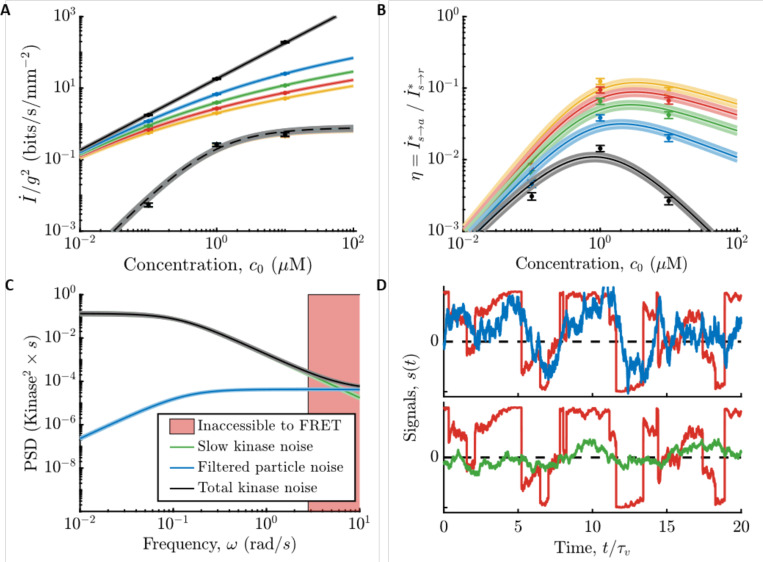
Comparing *E. coli*’s information rates to the particle counting limit. **A)** Information rates per gradient steepness squared, $g^{2}$, in particle arrivals, ${\dot{I}}_{s\to r}^{*}$ (SI Eqn. 44; solid lines), and in kinase activity, ${\dot{I}}_{s\to a}^{*}$ (SI Eqn. 89; dashed lines use Eqn. 16 and parameters measured in $c_{0}=1\;\text{μM}$) for gradients of varying steepness, $g\in \left\{0^{+},0.1,0.2,0.3,0.4\right\}\;{\text{mm}}^{-1}$ in black, blue, green, red, yellow, where black is the small gradient limit, g→0. Dots are experimental measurements. Error bars and shading throughout are SEM. We find that *E. coli* far from the physical limit set by particle arrivals when signals are weak and sensor quality matters. In particular, the fundamental limit ${\dot{I}}_{s\to r}^{*}$ scales slower than $g^{2}$, even for moderate g, indicating that it is out of the small-signal regime. Information in kinase activity ${\dot{I}}_{s\to a}^{*}$, on the other hand, is roughly proportional to $g^{2}$ (the lines are on top of each other), indicating that *E. coli* are still in the small-signal regime. **B)** $\eta ={\dot{I}}_{s\to a}^{*}/{\dot{I}}_{s\to r}^{*}$ versus $c_{0}$. Colors and markers are same as in (A). In steeper gradients, the quality of *E. coli*’s chemosensory apparatus matters less for getting close to the limit. **C)** Fit models for the noise power spectra in background concentration $c_{0}=1\;\text{μM}$. Green: fit to measured slow noise in kinase activity. Blue: particle arrival noise filtered through kinase response kernel. Black: Sum of green and blue, used as a conservative estimate of information in kinase activity. Red shading: experimentally-inaccessible region using CheY-CheZ FRET. See also SI Fig. S3 and the SI section “Modeling kinase activity” for discussion about noise in the red region. If *E. coli* were close to the physical limit, the black line would be close to the blue line at all frequencies. Instead, excess slow noise in kinase activity dominates over the entire range of observable frequencies. **D)** *E. coli*’s low information rates relative to the physical limit correspond to poor estimates of the signal s(t). Red: true signal from Fig. 1 with $c_{0}=1\;\text{μM}$ and $g=0.3\;{\text{mm}}^{-1}$. Top, blue: reconstructed signal from particle arrival rate r in Fig. 1, using the optimal causal kernel (SI Eqn. 57). Bottom, green: reconstructed signal from kinase activity a in Fig. 1, using the optimal causal kernel (SI Eqn. 95).

Both *E. coli*’s information rate, ${\dot{I}}_{s\to a}^{*}$, and the physical limit, ${\dot{I}}_{s\to r}^{*}$, are approximately proportional to the gradient steepness squared, $g^{2}$ in the limit of a shallow gradient (black lines in Fig. 3AB). Therefore, we quantify the information rates per $g^{2}$, using the parameters measured in the previous section. In particular, we plot the full expressions for the information rates, which are given in the SI. In Fig. 3A, we plot these quantities as functions of background concentration $c_{0}$, for varying values of the gradient steepness $g\in [0,0.4]\;{\text{mm}}^{-1}$, within which we observed linear dependence of chemotaxis drift speed on g (1). Doing so reveals that *E. coli* are surprisingly far from the physical limit: in shallow gradients, ${\dot{I}}_{s\to a}^{*}$ is at least two orders of magnitude below ${\dot{I}}_{s\to r}^{*}$ across all background concentrations.

To quantify the fidelity of *E. coli*’s chemical sensing relative to the physical limit, we computed the ratio of *E. coli*’s information rate relative to the physical limit, $\eta \equiv \frac{{\dot{I}}_{s\to a}^{*}}{{\dot{I}}_{s\to r}^{*}}$. We first focus on the limit of vanishingly small gradients, where η is independent of g, and we plot it in Fig. 3B (black) as a function of background concentration, $c_{0}$. In low backgrounds, $c_{0}\ll K_{i}$, the kinase signal-to-noise ratio, $\gamma_{a}$, scales as $c_{0}^{2}$ since *E. coli*’s gain $G_{r}$ and noise in kinase activity are constant. Thus, *E. coli*’s information rate scales as ${\dot{I}}_{s\to a}^{*}\propto c_{0}^{2}$. Since the physical limit scales as ${\dot{I}}_{s\to r}^{*}\propto c_{0}$, we get $\eta \propto c_{0}$, which goes to zero with decreasing background concentration. In high backgrounds, $c_{0}\gg K_{i}$, the kinase signal-to-noise ratio $\gamma_{a}$ is approximately constant because the gain depends on background concentration as $G_{r}\propto 1/c_{0}$, which cancels the concentration-dependence of the molecular arrival rate, $r_{0}\propto c_{0}$, and so ${\dot{I}}_{s\to a}^{*}$ is constant. As a result, we get $\eta \propto 1/c_{0}$, which again goes to zero with increasing concentration. These two regimes are separated by a peak at $c_{0}=K_{i}$, where η≈0.014±0.002 at our closest measured data point (black in Fig. 3B). In this background, the variance of filtered particle arrival noise is largest, but it is still much smaller than the variance of other kinase noise sources (see Figs. S1, S3).

For small but finite gradients, we find that η increases as the gradient g gets steeper, increasing to η≈0.1 when $g=0.4\;{\text{mm}}^{-1}$. This smaller value of η does not imply that *E. coli* count every particle in steeper gradients. Instead, η increases with g because the information rate, ${\dot{I}}_{s\to r}^{*}$, saturates in steeper gradients (solid color lines decreasing with g in Fig. 3A). In a steep gradient, even a poor sensor can accurately infer the signal, s(t), and increasing particle counts only provides marginal gains on the information rate. Mathematically, this can be seen through the weak dependence of ${\dot{I}}_{s\to r}^{*}$ on g outside of the small-signal regime (Fig. 3A). ${\dot{I}}_{s\to a}^{*}$, on the other hand, remains roughly proportional to $g^{2}$ to much steeper gradients. Thus, kinase activity is still in the small-signal regime in conditions where particle arrivals are not. In steeper gradients where signal can be reconstructed accurately, *E. coli* are able to get closer to the information bound even with a sensor that is far from counting every particle.

We support this further in Figs. 3CD. In Fig. 3C, we show the power spectrum of total noise in kinase activity (green line) compared to the power spectrum of filtered particle arrival noise (blue line). If *E. coli* were close to the particle-counting limit, nearly all noise in kinase activity would come from filtering particle arrivals; instead, kinase fluctuations are much larger over the range of frequencies observable in experiment (Fig. 3C, outside the pink region). We extrapolate to higher frequencies by conservatively assuming that the lines approach each other (black line), but it is possible that there are additional high frequency noise sources (putting the black line higher in shaded region of Fig 3C) or that the response function has a slower $\tau_{1}$ than in our model (putting the blue line lower in pink shaded region of Fig 3C). The information rate is relatively insensitive to these choices (see SI Fig S3 for discussion). In Fig. 3D, we show the optimal reconstructions of s(t) in Fig. 1, both from past particle arrivals {r} and from past kinase activity {a} using the parameter values determined from the experiments. The fidelity of the reconstruction from kinase activity is visibly worse than that from particle arrivals, consistent with the much lower information about the signal encoded in the kinase activity. Thus, *E. coli*’s information about signals during chemotaxis is not limited by the physical limit set by counting single particle arrivals.

## Discussion

Here, we studied how the physics of chemosensing (2) limits *E. coli*’s ability to encode information about signals relevant for chemotaxis. We derived a physical limit on information about the current time derivative of concentation, which we previously showed cells need for chemotaxis (1), by considering an ideal sensor able to register the arrival of every particle at its surface. We then measured the rate at which *E. coli* encode this information into the activity of their receptor-associated kinases through a series of single-cell measurements in multiple background concentrations of attractant. We found that *E. coli* are far from the physical limit of an idealized sensor, getting only a few percent of the information available in ligand particle arrivals in shallow gradients. Thus, the fidelity of *E. coli*’s chemosensing, and hence their chemotaxis performance, is not limited by the physics of molecule counting.

Previous work anticipated that *E. coli* would be much closer to the particle counting limit. Berg and Purcell argued that, in *E. coli* and *Salmonella typhimurium* chemotaxis, the change in concentration over a single run in a typical gradient could be estimated by an ideal agent with uncertainty smaller than the mean (2). From this, they concluded that the bacterial chemotaxis machinery is nearly optimal. However, their calculation does not imply that bacteria actually achieve that level of accuracy. Ref. (8) fit agent-based simulations to experimental measurements of *Vibrio ordalii* climbing dynamic chemical gradients and argued that this bacterium is within a factor of ~6 of the particle counting limit. However, this analysis assumed that cells infer s(t) in short, independent time windows of duration T=0.1s. Instead, real cells continuously monitor new particle arrivals and forget old ones, allowing them to average out molecule counting noise for integration times up to the signal correlation time $\tau_{v}$. This increases the theoretical maximum precision in the analysis of Ref. (8), and thus *V. ordalii*’s distance from the limit, by a factor of ${\left(\tau_{v}/T\right)}^{3}={\left(\frac{0.45\;s}{0.1\;s}\right)}^{3}∼90$, due to the $T^{3}$ in the uncertainty about signal (35). We believe this explains the discrepancy between our findings. It also suggests that similar constraints might limit the sensing fidelity of *E. coli* and other bacterial species.

We discovered a new relationship between two previously-disconnected information quantities: the transfer entropy rate (40) and the predictive information (42). While past work has argued that signaling networks should carry predictive information (12,13,42,44,45), here we identify a specific behavior where performance depends quantitatively on a predictive information rate. This new predictive information rate allows us to distinguish two possible sources of inefficiency that we could not separate in our previous study (1). First, kinases could encode information about past signals 𝑠, which do not contribute to gradient climbing; and second, relevant information could be lost in communication with the motors. Using ${\dot{I}}_{s\to a}^{*}$ derived here, which isolates information about the present signal, we estimate that about 90% or more of the cell’s information rate to kinase activity is relevant to chemotaxis, depending on $c_{0}$ (see SI), implying that the remaining losses are in communication with the motor.

Our analysis has implications for how we think about intermediary variables in signal transduction pathways. While behavioral decisions often require information about a current (or possibly future) external signal, intermediate variables do not need to represent these in their current value. For example,the entire past trajectory of kinase activity, {a}, contains more information than its current value, a, about the current signal, s. This information can be extracted by downstream processing, all the way down to the motors (see SI section “Optimal kernel for estimating signal from kinase activity”). The information available to downstream processing is quantified by the predictive information rate, and critically, this quantity is agnostic to that processing. Here we took advantage of this property to measure the fidelity of the kinases without assuming their activity is an instantaneous, noisy readout of signal.

Why are *E. coli* so far from the particle counting limit? It may be that design constraints prevent them from reaching this limit. *E. coli* must be able to perform chemotaxis over many orders of magnitude in background concentration, which might impose trade-offs that prevent the system from achieving optimality. Fold-change detection enables this (79–81), but also causes *E. coli*’s gain, $G_{r}$, to decrease with increasing concentration (Methods). Thus, just to keep η from decreasing with $c_{0}$, *E. coli* would need to have kinase noise variance that decreases with concentration like $1/c_{0}$. Instead, we find that it is roughly constant. Suppressing fluctuations or amplifiying signals generally requires spending energy or resources (10–16,86,87), and those costs might not be worth the fitness benefit in this case. The mechanism of amplification is not well understood, but recent work has argued that it consumes energy (87–89). Thus, energetic and mechanical constraints might provide currently-unknown bounds on *E. coli*’s sensory fidelity.

Surely, *E. coli* have evolved under selection pressures other than climbing shallow gradients of aspartate. *E. coli* need to sense multiple ligands, such as amino acids, sugars, and peptides (62,90), some of which require different receptor types. But the presence of multiple receptor types in the receptor array reduces the cooperativity to any one ligand (74), while likely still contributing to signaling noise. *E. coli* may be under selection pressure not only to climb gradients but also to stay close to concentration peaks (18,19,92,93). Furthermore, we do not know the typical gradient steepness they have been selected to climb effectively. In an infinitely shallow gradient, we showed that an ideal sensor would allow a bacteria to climb gradients at least 10 times faster than typical *E. coli* (due to $I_{s\to a}^{*}/I_{s\to r}^{*}\approx 0.01$ and $v_{d}\propto {\left(I_{s\to a}^{*}\right)}^{1/2}$ (1)). However, in steeper gradients, where even a poor sensor can adequately measure direction, these gains would be far smaller. For example, in a relatively steep 500-micron gradient and background of 1μM of attractant, we estimate that a typical cell would get ~37% of the relevant information available to an ideal sensor, and could climb ~60% as fast. It may be that the typical gradients that have driven the evolution of *E. coli’s* sensory apparatus are sufficiently steep as to obviate the need for an ideal single-molecule sensor. In the laboratory, the amino acid gradients *E. coli* perceive when migrating collectively are typically of order ~1 mm (93), and theory predicts that they can be steeper in semisolid agar (94,95) in which our laboratory strain of *E. coli* was selected for chemotaxis (96–98).

Existing findings give qualitative support for the idea that *E. coli* are not at the fundamental limit. Berg and Purcell’s original paper argued that by evenly-distributing small, sparse receptors on its surface, a cell can make its ligand sensor nearly as effective as if its entire surface were covered with receptors (2). Thus, a chemosensor limited primarily by the noise of single particle arrivals would want to spread a limited receptor budget evenly over the cell surface to maximize the rate at which unique particles are counted. Instead, bacterial chemoreceptors are clustered in densely-packed arrays. This dense packing, which appears to be universal across species (99), might be necessary for bacteria to integrate and amplify signal that must be communicated to the motor to make all-or-none behavioral decisions.

Future experiments could probe whether hard constraints prevent *E. coli* from being close to the physical limit, or if tradeoffs would allow a cell to do better, perhaps at the cost of increased energy expenditure. This could be done by measuring information rates in single cells, where cell-to-cell variability (63,66–68,70,72,78,101,102) might enable some cells to be closer to the physical limit by chance.

While *E. coli* do not achieve the particle counting bound, their sensory capabilities are impressive. In the log-sensing regime they aquire and communicate information to the motor at a rate equivalent to an ideal sensor able to count several thousand particles every second. While current modeling efforts in chemosensing have mostly focused on quantitatively describing experimental observations, this work opens up new possibilities for a reverse engineering perspective. Our work highlights the need to understand how these systems achieve the signal processing, bandwidth, and fidelity needed for behavior, and how physical, geometric, and energetic constraints have shaped their evolution.

## Methods

### Modeling of average kinase responses to past signal versus past particle arrival rate

In our previous work (1), we modeled responses of kinase activity to past signals s instead of past particle arrival rate r. These two descriptions are equivalent in the regime of shallow gradients. We show this below by starting from average responses of kinase activity to particle arrival rate:

(10) $$\langle a(t)\rangle =a_{0}-\int_{-\infty }^{t}K_{r}\left(t-t^{\prime }\right)\left(\langle r\left(t^{\prime }\right)\rangle -r_{0}\right)\;dt^{\prime },$$

where angled brackets indicate averaging over repeated presentation of the same signal trajectory {s}, and thus they average out particle noise and kinase noise. From here, we will derive a response kernel to past signals that gives identical kinase responses.

First, we note that:

(11) $$\langle r(t)\rangle -r_{0}=k_{D}\left(c(t)-c_{0}\right)=r_{0}\int_{-\infty }^{t}s\left(t^{\prime }\right)\;dt^{\prime },$$

where we used $s(t)\approx \frac{1}{c_{0}}\frac{dc}{dt}$ in shallow gradients.

It is convenient to transform the expressions above to Fourier space, where $\delta a(\omega )=F\left[\langle a(t)\rangle -a_{0}\right]$, $\delta r(\omega )=F\left[\langle r(t)\rangle -r_{0}\right]$, $K_{r}(\omega )=F\left[K_{r}(t)\right]$, and $F[f(t)]=\int_{-\infty }^{\infty }f\;(t)\;e^{i\;\omega \;t}\;dt$ is the Fourier transform.

Then we have

(12) $$\delta a(\omega )=-K_{r}(\omega )\;\delta r(\omega ),$$

(13) $$\delta r(\omega )=r_{0}\frac{s(\omega )}{-i\;\omega }.$$

With this, we get:

(14) $$\delta a(\omega )=-K_{r}(\omega )\;r_{0}\frac{s(\omega )}{-i\omega }=-K(\omega )\;s(\omega )$$

where $K(\omega )=r_{0}\frac{K_{r}(\omega )}{-i\omega }$ is the Fourier transform of the linear response function to signals. Thus, we can either write down average kinase responses to particle arrival rate r(t), with linear response function $K_{r}(t)$, or responses to signals s(t), with linear response function K(t) (1):

(15) $$K(t)=r_{0}\int_{0}^{t}K_{r}\left(t^{\prime }\right)\;dt^{\prime }=G\;\exp\left(-\frac{t}{\tau_{2}}\right)\;\left(1-\exp\left(-\frac{t}{\tau_{1}}\right)\right).$$

where we have defined the MWC model gain $G=r_{0}\;G_{r}$ (23,76). Thus:

(16) $$G_{r}=\frac{1}{r_{0}}G\approx \frac{1}{k_{D}}\frac{G_{\infty }}{c_{0}+K_{i}}.$$

We can use the response function to particle arrivals, $K_{r}(t)$, to compute the power spectrum of particle counting noise filtered through the kinase response kernel, $K_{r}(t)$, but expressed it in terms of the response kernel K(t) to signals s. Since we model particle arrival noise as shot noise, its power spectrum is constant and equal to $r_{0}$. Filtering this noise through the response kernel $K_{r}(\omega )$ gives:

(17) $$N_{r}(\omega )=r_{0}{\left|K_{r}(\omega )\right|}^{2}=r_{0}{\left|\frac{-i\;\omega }{r_{0}}K(\omega )\right|}^{2}=\frac{1}{r_{0}}\omega^{2}|K(\omega ){|}^{2}.$$

### Simulation details in Figure 1

Simulation time step was $dt=3\times 10^{-3}\;\tau_{v}$. Signal s(t) was simulated in 2D by randomly sampling the times of instantaneous tumbles, plus rotational diffusion during runs, which was implemented using the Euler-Maruyama method. Average particle arrival rate 〈r(t)〉 was computed from the signal, and then Gaussian noise of variance $\sqrt{r_{0}\;dt}$ was added to mimic shot noise. Kinase activity a(t) was simulated using the model in the main text (Eqn. 6), with biologically reasonable parameters (see Fig. 2).

### Strains and plasmids

All strains and plasmids used are the same as in our recent work (1). The strain used for the FRET experiments is a derivative of *E. coli* K-12 strain RP437 (HCB33), a gift of T. Shimizu, and described in detail elsewhere (59,70). The FRET acceptor-donor pair (CheY-mRFP and CheZ-mYFP) is expressed in tandem from plasmid pSJAB106 (59) under an isopropyl β-D-thiogalactopyranoside (IPTG)-inducible promoter. The glass-adhesive mutant of FliC (FliC*) was expressed from a sodium salicylate (NaSal)-inducible pZR1 plasmid (59). The plasmids are transformed in VS115, a *cheY cheZ fliC* mutant of RP437 (59) (gift of V. Sourjik). RP437, the direct parent of the FRET strain and also a gift from T. Shimizu, was used to measure swimming statistics parameters. All strains are available from the authors upon request.

### Cell preparation

Single-cell FRET microscopy and cell culture was carried out essentially as described previously (1,59,70,72). Cells were picked from a frozen stock at −80°C and inoculated in 2 mL of Tryptone Broth (TB; 1% bacto tryptone, 0.5 % NaCl) and grown overnight to saturation at 30°C and shaken at 250 RPM. Cells from a saturated overnight culture were diluted 100X in 10 mL TB and grown to OD600 0.45–0.47 in the presence of 100 μg/ml ampicillin, 34 μg/ml chloramphenicol, 50 μM IPTG and 3 μM NaSal, at 33.5°C and 250 RPM shaking. Cells were collected by centrifugation (5 min at 5000 rpm, or 4080 RCF) and washed twice with motility buffer (10 mM KPO4, 0.1 mM EDTA, 1 μM methionine, 10 mM lactic acid, pH 7), and then were resuspended in 2 mL motility buffer, plus the final concentration of Asp. Cells were left at 22°C for 90 minutes before loading into the microfluidic device. All experiments, FRET and swimming, were performed at 22–23°C.

For swimming experiments, cells were prepared similarly. Saturated overnight cultures were diluted 100X in 5 mL of TB. After growing to OD600 0.45–0.47, 1 mL of cell suspension was washed twice in motility buffer with 0.05% w/v of polyvinylpyrrolidone (MW 40 kDa) (PVP-40). Washes were done by centrifuging the suspension in an Eppendorf tube at 1700 RCF (4000 RPM in this centrifuge) for 3 minutes. After the last wash, cells were resuspended with varying background concentrations of Asp.

### Microfluidic device fabrication and loading for FRET measurements

Microfluidic devices for the FRET experiments (70–72) were constructed from polydimethylsiloxane (PDMS) on 24 × 60 mm cover glasses (#1.5) following standard soft lithography protocols (102), exactly as done before (1).

Sample preparation in the microfluidic device was conducted as follows. Five inlets of the device were connected to reservoirs (Liquid chromatography columns, C3669; Sigma Aldrich) filled with motility buffer containing various concentrations of Asp through polyethylene tubing (Polythene Tubing, 0.58 mm id, 0.96 mm od; BD Intermedic) (see SI of (1)). The tubing was connected to the PMDS device through stainless steel pins that were directly plugged into the inlets or outlet of the device (New England Tubing). Cells washed and suspended in motility buffer were loaded into the device from the outlet and allowed to attached to the cover glass surface via their sticky flagella by reducing the flow speed inside the chamber. The pressure applied to the inlet solution reservoirs was controlled by computer-controlled solenoid valves (MH1; Festo), which rapidly switched between atmospheric pressure and higher pressure (1.0 kPa) using a source of pressurized air. Only one experiment was conducted per device. *E. coli* consume Asp, so all experiments below were performed with a low dilution of cells to minimize this effect. The continuous flow of fresh media also helped ensured that consumption of Asp minimally affected the signal cells experienced.

### Single-cell FRET imaging system

FRET imaging in the microfluidic device was performed using the setup as before (1), on an inverted microscope (Eclipse Ti-E; Nikon) equipped with an oil-immersion objective lens (CFI Apo TIRF 60X Oil; Nikon). YFP was illuminated by an LED illumination system (SOLA SE, Lumencor) through an excitation bandpass filter (FF01–500/24–25; Semrock) and a dichroic mirror (FF520-Di02–25×36; Semrock). The fluorescence emission was led into an emission image splitter (OptoSplit II; Cairn) and further split into donor and acceptor channels by a second dichroic mirror (FF580-FDi01–25×36; Semrock). The emission was then collected through emission bandpass filters (F01–542/27–25F and FF02–641/75; Semrock; Semrock) by a sCMOS camera (ORCA-Flash4.0 V2; Hamamatsu). RFP was illuminated in the same way as YFP except that an excitation bandpass filter (FF01–575/05–25; Semrock) and a dichroic mirror (FF593-Di03–25×36; Semorock) were used. An additional excitation filter (59026x; Chroma) was used in front of the excitation filters. To synchronize image acquisition and the delivery of stimulus solutions, a custom-made MATLAB program controlled both the imaging system (through the API provided by Micro-Manager (103)) and the states of the solenoid valves.

### Computing FRET signal and kinase activity

FRET signals were extracted from raw images using the E-FRET method (104), which corrects for different rates of photobleaching between donor and acceptor molecules. In this method, YFP (the donor) is illuminated and YFP emission images $\left(I_{DD}\right)$ and RFP (the acceptor) emission images $\left(I_{DA}\right)$ are captured. Periodically, RFP is illuminated and RFP emission images are captured $\left(I_{AA}\right)$. From these, photobleach-corrected FRET signal is computed as before (1), which is related to kinase activity a(t) by an affine transform when CheY and CheZ are overexpressed (1,73). All parameters associated with the imaging system were measured previously (1).

In each experiment, we first delivered a short saturating stimulus (1 mM MeAsp plus 100 μM serine (74)) to determine the FRET signal at minimum kinase activity, followed by motility buffer with Asp at background concentration $c_{0}$. Before the saturating stimulus was delivered, the donor was excited every 0.5 seconds to measure $I_{DD}$ and $I_{DA}$ (see SI of (1)) for 5 seconds. Then the stimulus was delivered for 10 seconds, and the donor was excited every 0.5 seconds during this time. Before and after the donor excitations, the acceptor was excited three times in 0.5-second intervals to measure $I_{AA}$ (see SI of (1)). After the stimulus was removed, the acceptor was excited three more times at 0.5-second intervals. Imaging was then stopped and cells were allowed to adapt to the background for 120 seconds.

Stimulus protocols for measuring kinase linear response functions and fluctuations are described below.

At the end of each experiment, we delivered a long saturating stimulus (1 mM MeAsp plus 100 μM serine) for 180 seconds to allow the cells to adapt. Then we removed the stimulus back to the background concentration, eliciting a strong response from the cells, from which we determined the FRET signal at maximum kinase activity. The donor was excited for 5 seconds before the saturating stimulus and 10 seconds after it, every 0.5 seconds. Before and after these donor excitations, the acceptor was excited three times in 0.5-second intervals. The cells were exposed to the saturating stimulus for 180 seconds. The donor was excited every 0.5 seconds for 5 seconds before cells were exposed to motility buffer with Asp at background concentration $c_{0}$, followed by 10 seconds of additional donor excitations. Before and after the donor excitations, the acceptor was again excited three times in 0.5-second intervals.

FRET signals were extracted as before (1). The FRET signal at minimum kinase activity, $FRET_{min}$, was computed from the average FRET signal during the first saturating stimulus. The FRET signal at maximum kinase activity, $FRET_{max}$, was computed from the average FRET signal during the first quarter (2.5 seconds) of the removal stimulus at the end of the experiment. Kinase activity was then computed from corrected FRET signal: $a(t)=\frac{FRET(t)-FRET_{min}}{FRET_{max}-FRET_{min}}$.

### Kinase linear response functions

Experiments were performed in Asp background concentrations $c_{0}$ of 0.1, 1, and 10 μM. Measurements were made in single cells, and at least three replicates were performed per background. FRET level at minimum kinase activity was measured at the beginning of each experiment, as described above. After this, a series of stimuli were delivered to the cells in the microfluidic device. Cells were only illuminated and imaged when stimulated in order to limit photobleaching. Before each stimulus, cells were imaged for 7.5 seconds in the background concentration $c_{0}$. Then, the concentration of Asp was shifted up to $c_{+}>c_{0}$ for 30 seconds and imaging continued. Donor excitation interval was 0.75 seconds and acceptor excitations were done before and after the set of donor excitations. After this, imaging was stopped and the Asp concentration returned to $c_{0}$ for 65 seconds to allow cells to adapt. Then, the same process was repeated, but this time shifting Asp concentration down to $c_{-}<c_{0}$. Alternating up and down stimuli were repeated 10 times each. $c_{+}$ and $c_{-}$ varied with each experiment and each background $c_{0}$. Finally, FRET level at maximum kinase activity was measured at the end of each experiment, as described above. The whole imaging protocol lasted <2200 seconds. In total, cells spent <60 minutes in the device, from loading to the end of imaging.

These data were analyzed as before (1) to extract linear response parameters for each cell. In brief, the responses of a cell to all steps up or steps down in concentration were averaged and the standard error of the response at each time point computed. Model parameters were extracted by maximizing the posterior probability of parameters given data, assuming a Gaussian likelihood function and log-uniform priors for the parameters. The uncertainties of single-cell parameter estimates were generated by MCMC sampling the posterior distribution. Finally, the population-median parameters were computed from all cells in experiments in a given background $c_{0}$. Uncertainty $\sigma_{\theta_{i}}^{2}$ of the population-median value of parameter $\theta_{i}$, with $\theta =\left(G,\tau_{1},\tau_{2}\right)$, was computed using:

(18) $$\sigma_{\theta_{i}}^{2}=\frac{1}{N}{\left(1.4826\;\text{mad}\left(\left\{\theta_{i}^{MAP}\right\}\right)\right)}^{2}+\frac{1}{N^{2}}\sum_{k}{\left(\sigma_{\theta_{i}}^{2}\right)}_{k}.$$

This expression accounts both for cell-to-cell variations (first term) and uncertainties in the single-cell estimates (second term). N is the number of cells. 1.4826 mad( ) is an outlier-robust uncertainty estimate that coincides with the standard deviation when the samples are Gaussian-distributed, and mad( ) is the median absolute deviation, used previously (1). $\left\{\theta_{i}^{MAP}\right\}$ are the single-cell maximum *a-posteriori* (MAP) estimates of parameter $\theta_{i}$. ${\left(\sigma_{\theta_{i}}^{2}\right)}_{k}$ is the uncertainty of $\theta_{i}^{MAP}$ in cell k, which was computed using

(19) $${\left(\sigma_{\theta_{i}}\right)}_{k}=1.4826\;\text{mad}\left({\left\{{\hat{\theta }}_{i}\right\}}_{k}\right)$$

where ${\left\{{\hat{\theta }}_{i}\right\}}_{k}$ are the samples from the kth cell’s posterior via Markov Chain Monte Carlo (MCMC).

### MWC kinase gain

The estimated gain parameter G depended strongly on $c_{0}$, consistent with expectations from previous work modeling kinase activity using the MWC model (e.g. (76)). In the MWC model, kinase-receptor complexes can be in active or inactive states. The dissociation constants for the attractant in each state, $K_{i}$ and $K_{a}$, are different, with $K_{i}\ll K_{a},$ which causes attractant concentration to influence the fraction of kinases in the active state. When the background concentration $c_{0}\ll K_{a}$, the gain of the kinase response to changes in log-concentration of attractant can be written:

$$G\left(c_{0}\right)=G_{\infty }\frac{c_{0}}{c_{0}+K_{i}},$$

where $G_{\infty }$ is the “log-sensing” gain (when $c_{0}\gg K_{i}$). Parameters $G_{\infty }$ and $K_{i}$ were estimated by fitting the estimates of G versus $c_{0}$. The fit was done by minimizing the sum of squared errors between the logarithms of G and $G_{MWC}$. The estimated values of G vary by about an order of magnitude, and taking the logarithms ensured that the smallest value of G had similar weight as largest value in the objective function.

### Statistics of noise in kinase activity

Fluctuations in kinase activity were measured in the same Asp background concentrations $c_{0}$ as above, as well as $c_{0}=0\;\text{μM}$. At least three replicate experiments were performed per background. FRET level at minimum kinase activity was measured at the beginning of each experiment, as described above. After these measurements, imaging was then stopped and cells were allowed to adapt to the background for 120 seconds. After this, cells were imaged for about 1200 seconds. Throughout, donor excitations were done every 1.0 second, except when it was interrupted by acceptor excitations, which were conducted every 100 donor excitations (see SI of (1)). Finally the FRET level at maximum kinase activity was measured at the end of each experiment, as described above. The whole imaging protocol lasted <1400 seconds. In total, cells spent about < 60 minutes in the device, from loading to the end of imaging.

These data were analyzed as before (1). Bayesian filtering methods (82) were used to compute the likelihood of the parameters given the data, and the prior distribution was taken to be uniform in log. Single-cell estimates and uncertainties of the noise parameters were extracted from the posterior distribution as described above. In each background $c_{0}$, the population median parameter values were computed, and their uncertainties were computed as described above, with $\theta =\left(D_{n},\tau_{n}\right)$.

### Swimming velocity statistics

Cells were prepared and imaged as before (1). After the second wash step of the Cell preparation section above, cells were centrifuged again and resuspended in motility buffer containing a background concentration of Asp $c_{0}$. The values of $c_{0}$ used here were the same as in the FRET experiments, including $c_{0}=0\;\text{μM}$. Then, the cell suspension was diluted to an OD600 of 0.00025. This low dilution of cells both enables tracking and minimizes the effect of cells consuming Asp. The cell suspension was then loaded into μ-Slide Chemotaxis devices (ibidi; Martinsried, Germany). Swimming cells were tracked in one of the large reservoirs. 1000-s movies of swimming cells were recorded on a Nikon Ti-E Inverted Microscope using a CFI Plan Fluor 4X objective (NA 0.13). Images were captured using a sCMOS camera (ORCA-Flash4.0 V2; Hamamatsu). Four biological replicates were performed for each background $c_{0}$.

Cell detection and tracking were carried out using the same custom MATLAB as we used previously (1), with the same analysis parameters (see SI of that paper for details). Tumble detection was also carried out identically as before (1). There was no minimum trajectory duration, but cells were kept only if at least two tumbles were detected in their trajectory. For each cell, we computed the fraction of time spent in the “run” state $P_{run}$. Then we constructed the distribution of $P_{run}$, correcting for biases caused by the different diffusivities of cells with different $P_{run}$ (1). As before (1), we then computed the correlation function of velocity along one spatial dimension for each cell, $V_{i}(t)={\langle v_{x}\left(t^{\prime }\right)v_{x}\left(t^{\prime }+t\right)\rangle }_{t^{\prime }}$. among cells with $P_{run}$ within ±0.01 of the population-median value,. Finally, we computed a weighted average of the correlation functions over all cells in the population-median bin of $P_{run}$, where trajectories were weighted by their duration, giving V(t). In each background $c_{0}$, for the median bin of $P_{run}$, the average trajectory duration was ~7.6 seconds, and the total trajectory time was ≥ 2.7 × 10^4^ seconds.

These correlation functions V(t) in each background $c_{0}$ and each experiment were fit to decaying exponentials $\sigma_{v}^{2}\exp\left(-|t|/\tau_{v}\right)$, and the parameters and their uncertainties were extracted in two steps. First, we determined the MAP estimates of the parameters. An initial estimate of the parameters were esimated using the MATLAB *fit* function to fit exponentials to the V(t) in the time rang t∈[2Δt,10s], with Δt=50ms. The estimated $\tau_{v}$ was used to get the uncertainty of V(t) in each experiment, as done before (1). Assuming a Gaussian likelihood function and parameters distributed uniformly in logarithm, the posterior distribution of parameter was constructed. In each experiment, MAP estimates of the parameters were extracted as done for the kinase parameters, and parameter uncertainties were computed from MCMC samples of the posterior distribution as above. Finally, we computed the average parameters $\sigma_{v}^{2}$ and $\tau_{v}$ over experimental replicates, as well as their standard errors over replicates.

### Additional error analysis

Once the variance of the population-median value of parameter i was computed, $\sigma_{\theta_{i}}^{2}$, we propagated the uncertainty to functions of those parameters. For some function of the parameters, f(θ), we computed the variance of f(θ), $\sigma_{f}^{2}$, as:

$$\sigma_{f}^{2}=\sum_{i}{\left(\frac{\partial f}{\partial \theta_{i}}\right)}^{2}\sigma_{\theta_{i}}^{2}$$

(20) $$=f^{2}\sum_{i}{\left(\frac{\partial \log f}{\partial \theta_{i}}\right)}^{2}\sigma_{\theta_{i}}^{2}.$$

The equations above neglect correlations in the uncertainties between pairs of parameters. This was used to compute the uncertainties of ${\dot{I}}_{s\to r}^{*}$, ${\dot{I}}_{s\to a}^{*}$, and η. The same formula was used to compute uncertainties of functions of time by applying the formula above pointwise at each time delay t and neglecting correlations in uncertainties between time points.

## Supplementary Material

Supplement 1

## Data Availability

Source data for the main text figures are provided online with the manuscript. Source data for the Supplementary Figures are contained in a Supplementary Data file.
