# Chemotaxing *E. coli* do not count single molecules

**Published:** 2024-11-27

**Authors:** Henry H. Mattingly, Keita Kamino, Jude Ong, Rafaela Kottou, Thierry Emonet, Benjamin B. Machta

**Affiliations:** 1 Center for Computational Biology, Flatiron Institute; 2 Institute of Molecular Biology, Academia Sinica; 3 Molecular, Cellular, and Developmental Biology, Yale University; 4 Physics, Yale University; 5 QBio Institute, Yale University

## Abstract

Understanding biological functions requires identifying the physical limits and system-specific constraints that have shaped them. In *Escherichia coli* chemotaxis, gradient-climbing speed is information-limited, bounded by the sensory information they acquire from real-time measurements of their environment. However, it remains unclear what limits this information. Past work conjectured that *E. coli*’s chemosensing is limited by the physics of molecule arrivals at their sensors. Here, we derive the physical limit on behaviorally-relevant information, and then perform single-cell experiments to quantify how much information *E. coli*’s signaling pathway encodes. We find that *E. coli* encode two orders of magnitude less information than the physical limit due to their stochastic signal processing. Thus, system-specific constraints, rather than the physical limit, have shaped the evolution of this canonical sensory-motor behavior.

## Introduction

Selection optimizes function, and therefore biological systems are shaped by complex fitness objectives and constraints. This has motivated using normative theories, subject only to constraints of physics, to derive fundamental limits on function and to predict the design of biological systems (1–17). This approach has been especially successful in the context of sensing, where theories of optimal estimation can be brought to bear. However, biology needs to implement sensing and other functions using non-ideal components, in the confines of a body, and with limited resources, introducing additional system-specific constraints (18–25). Understanding what bounds or constraints meaningfully limit biological functions would shed light on the forces that have shaped their evolution.

*Escherichia coli* chemotaxis is an ideal system for studying these limits on biological functions (26–28). *E. coli* climb chemical gradients by alternating between straight-swimming runs and randomly-reorienting tumbles (29). As they swim, they measure time-dependent concentrations of attractants, encode these measurements into the activity of intracellular CheA kinase activity, and use this information to decide when to tumble. Importantly, chemotaxis provides a fitness advantage, even above undirected motility, in structured chemical environments (30).

The accuracy with which a cell encodes its chemical environment can be quantified by an information rate. Given this information rate, we recently showed that there is a theoretical bound on how fast a cell can climb a gradient (17). Although typical *E. coli* cells get very little information—about 0.01 bits/s in a centimeter-long gradient—we found that they use it to climb gradients at speeds near the theoretical bound. Thus, *E. coli* chemotaxis is information-limited.

What prevents *E. coli* from obtaining more information during chemotaxis? Berg and Purcell demonstrated that the stochastic arrival of diffusing ligand molecules at a sensor fundamentally limits the accuracy of chemical sensing (4). Going a step further, they argued that bacteria approach this physical sensing limit, inspiring an entire field of biophysics (18,19,31–42). Recent experimental work claimed that a marine bacterium approaches this limit (43). However, it is still unclear whether this physical limit, as opposed to system-specific, internal limitations, meaningfully constrains chemosensing in *E. coli* and other bacteria. Answering this question has been challenging because of difficulties determining which external signals are relevant for chemotaxis, and then measuring and interpreting cells’ internal encodings of those signals.

Here, we address these challenges using a combination of information theory and single-cell FRET measurements. First, we derive the physical limit on the rate at which chemotactically-relevant information can be acquired, set by the stochasticity of molecule arrivals. Next, we derive an expression for the rate at which *E. coli* encode this information in their kinase activity. Using single-cell measurements, we quantify these information rates, finding that a typical *E. coli* cell gets orders of magnitude less information than the physical limit. In particular, signal transduction noise far exceeds molecule arrival noise; thus, *E. coli* chemotaxis is internally-limited. Our work raises questions about what specific constraints limit their chemosensing, and more broadly suggest that a combination of normative theories and system-specific details are required to understand the design of biological systems.

### Bacterial chemotaxis requires information about the current time derivative of concentration

**Figure 1:**
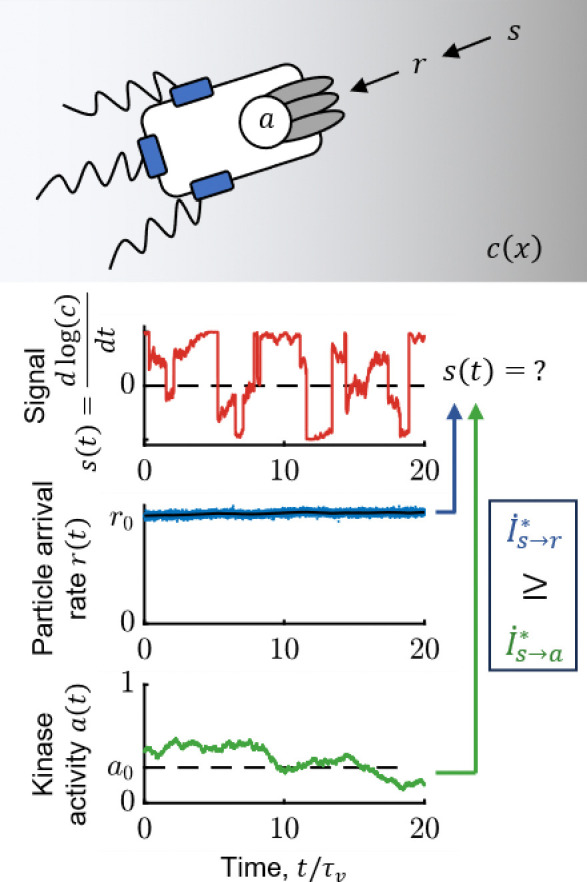
*E. coli* need to infer the rate of change of attractant concentration from noisy measurements. Top: Bacteria do not have a direct access to behaviorally-relevant signal $s=\frac{d}{dt}log(c)$—instead, they can at best measure molecules stochastically arriving with rate r(t) at their transmembrane receptors. Receptor-associated kinases respond to ligand arrivals with changes in activity, a(t), and encode information about s(t), but also introduce noise. Bottom: Simulated traces of s(t) (red); r(t) (blue); $\left\langle r(t)\rangle =k_{D}\;c(t\right)$ (black); and kinase activity a(t) (green) for a cell exhibiting run-and-tumble motion in a shallow chemical gradient. $r_{0}$ is the background particle arrival rate, $r_{0}=k_{D}\;c_{0}$, and $a_{0}$ is the baseline level of kinase activity. The cell’s task is to infer s(t) from past kinase activity {a}, and the accuracy of this inference is quantified by the information rate, ${\dot{I}}_{s\to a}^{*}$. An ideal agent would directly estimate s(t) from past molecule arrival rate {r}, thus setting the physical limit, $i_{s\to r}^{*}$. The simulation above was performed in a background concentration $c_{0}=1\;\mu M$ and gradient of steepness $g\;=\;0.3\;{mm}^{-1}$.

The goal of the chemotaxis system is not to estimate the current concentration per se, but instead to move up a chemical gradient. Therefore, cells need to capture behaviorally-relevant aspects of the chemical environment. We recently identified the behaviorally-relevant signal for *E. coli* chemotaxis to be the instanteous time derivative of (log) concentration, $s(t)=\frac{d}{dt}log(c)$ (Fig. 1) (17). Gradient-climbing speed is determined by a transfer entropy rate (44) that quantifies how much information about this signal, (t), is encoded in the past of kinase activity, {a} (SI):

(1) $${\dot{I}}_{s\to a}^{*}\equiv \underset{dt\to 0}{lim}\;\frac{1}{dt}I(a(t+dt);s(t)|\{a\}),$$

where I(X;Y|Z) is the mutual information between X and Y, conditioned on Z (45,46). Furthermore, due to the data-processing inequality and feed-forward structure of the chemotaxis pathway (46–48), the transfer entropy to any intermediate variable *bounds* information downstream, and thus bounds gradient-climbing speed.

In particular, the stochastic arrival rate of ligand molecules at the cell surface, r(t), is the first upstream quantity that encodes information about signal s(t) (Fig. 1). Thus, the transfer entropy to molecule arrival rate, ${\dot{I}}_{s\to r}^{*}$, sets the physical limit on the sensory information available for chemotaxis. An ideal agent would make navigation decisions based on a perfect readout of past particle arrivals {r}. By comparing the information about signals encoded in *E. coli*’s kinase activity, ${\dot{I}}_{s\to a}^{*}$, to the physical limit, ${\dot{I}}_{s\to r}^{*}$, we can determine whether their chemosensing is externally or internally limited.

### Physical limit on information due to stochastic molecule arrivals

We first derive an expression for the physical limit, ${\dot{I}}_{s\to r}^{*}$, from a model for the dynamics of s(t) and r(t). In static gradients, the signals a cell experiences are determined by their own run-and-tumble motion in the gradient. Accordingly, in a gradient of steepness g=dlog(c)/dx, the signal is $s(t)=gv_{x}(t)$, where $v_{x}$ is the cell’s up-gradient velocity. In shallow gradients (17,20), we can approximate s(t) as Gaussian with correlation function $\left\langle s(t)s\left(t^{\prime }\right)\right\rangle =g^{2}V\left(t-t^{\prime }\right)=g^{2}\sigma_{v}^{2}\;exp\;\left(-\frac{\left|t-t^{\prime }\right|}{\tau_{v}}\right)$. Here, V(t) is the correlation function of $v_{x}$ in the absence of a gradient, $\sigma_{v}^{2}$ is the variance of $v_{x}$, and $\tau_{v}$ is the signal correlation time, which depends on the cell’s mean run duration, the persistence of tumbles, and rotational diffusion (17,49).

Molecule arrival events follow a Poisson process with time-varying rate $k_{D}\;c\left(t\right)=4D\;l\;c(t)$, where $D\;\approx \;800\;\mu m^{2}/s$ (50,51) is the diffusivity of the ligand and l≈60nm (52) is the radius of a circular sensor on the cell’s surface (4,39). These give $k_{D}\approx 1.2\times 10^{5}{\;s}^{-1}\mu M^{-1}$, which is comparable to previous estimates (4,43). If many molecules arrive per run, $r_{0}\tau_{v}\gg 1$, we can approximate the Poisson process with a Gaussian process for the number of molecule arrivals per unit time, $r\left(t\right)=k_{D}\;c\left(t\right)+\sqrt{r_{0}}\;\xi (t)$. Here, $r_{0}=k_{D}\;c_{0}$ is the background molecule arrival rate, $c_{0}$ is the background concentration, and the noise is ⟨ξ(t)ξ(t′)⟩=δ(t-t′). We assume the sensor absorbs every molecule it senses (4), but if it cannot distinguish between new ligand arrivals and rebinding events, the limit is lower by an O(1) prefactor (39,40).

Since s(t) and {r} are approximately Gaussian, the physical limit has a simple form in terms of the variance of the optimal estimate of s(t) constructed from the past of $r,\sigma_{s|r}^{2}$ (SI Eqn. 29). This can be done using causal Wiener filtering theory (53–55) (see also (20,56–59)) (SI). We find that the physical limit on behaviorally-relevant information for chemotaxis in shallow gradients is:

(2) $${\dot{I}}_{s\to r}^{*}\approx \frac{1}{\tau_{v}}\frac{1}{4}\gamma_{r}.$$

Here, we defined the dimensionless signal-to-noise ratio of molecule arrivals, $\gamma_{r}=2\;r_{0}\;g^{2}\;\sigma_{v}^{2}\;\tau_{v}^{3}$. Eqn. 2 is valid when $\gamma_{r}\ll 1$, which sets the small-signal regime for ${\dot{I}}_{s\to r}^{*}$. We also provide a full expression for ${\dot{I}}_{s\to r}^{*}$ in the SI (SI Eqn. 60). Increasing the background $r_{0}$, the gradient steepness g, or the swimming speed $\sigma_{v}$ increases the signal-to-noise ratio of molecule arrivals. Longer runs, $\tau_{v}$, increase ${\dot{I}}_{s\to r}^{*}$ by allowing more time to average out noise. The derivation of ${\dot{I}}_{s\to r}^{*}$ also provides the optimal kernel for constructing a running estimate of s(t) from past molecule arrival rate {r}, which we discuss in the SI.

### Information encoded in *E. coli*’s CheA kinase activity

To derive the information encoded in CheA kinase activity, ${\dot{I}}_{s\to a}^{*}$, we next model kinase responses to molecule arrivals and noise. Kinase activity in shallow gradients can be modeled using linear response theory (17). For a cell with steady-state activity $a_{0}$ in background $r_{0}$:

(3) $$a(t)=a_{0}-\int_{-\infty }^{t}K_{r}\left(t-t^{\prime }\right)\left(r\left(t^{\prime }\right)-r_{0}\right)\;dt^{\prime }+\eta_{n}(t).$$

*E. coli* respond to a step increase in attractant concentration with a fast drop in kinase activity, followed by slow adaptation back to the pre-stimulus level (60). We model this phenomenologically with response function $K_{r}(t)=G_{r}\left(\frac{1}{\tau_{1}}exp\left(-\frac{t}{\tau_{1}}\right)-\frac{1}{\tau_{2}}exp\left(-\frac{t}{\tau_{2}}\right)\right)\;\Theta (t)$, where $G_{r}$ is the gain of the response to molecule arrival rate $r,\tau_{1}$ is the fast response time, $\tau_{2}$ is the slow adaptation time, and Θ(t) is the Heaviside step function. Kinase responses can equivalently be expressed in terms of past signals 𝑠, with a related kernel K(t) that we used previously (17) ($K_{r}(t)=\frac{1}{r_{0}}\frac{d}{dt}K(t)$; SI Eqn. 9).

Noise in kinase activity is driven by a combination of stochastic molecule arrivals and internally-driven fluctuations. Previous single-cell FRET experiments have observed large, slow fluctuations in kinase activity, $\eta_{n}(t)$, on a time scale of 10 s (17,61–63). These are well-described as Gaussian, with correlation function $\left\langle \eta_{n}(t)\;\eta_{n}(t^{\prime })\right\rangle =D_{n}\tau_{n}\;exp\left(-\frac{\left|t-t^{\prime }\right|}{\tau_{n}}\right)$. Here, $D_{n}$ is the diffusivity of internal noise in kinase activity, and $\tau_{n}$ is its correlation time. In addition, Eqn. 3 has additive noise arising from responses to molecule arrival noise. To date, it has not been possible to measure kinase fluctuations on time scales shorter than the CheY-CheZ relaxation time ($\tau_{1}$), but it cannot go below the level set by responses to molecule arrival noise. Thus, the phenomenological model above agrees with experiments at low frequencies while obeying known physics at high frequencies.

Evaluating $I_{s\to a}^{*}$ again reduces to deriving the variance of the estimated signal s(t) constructed from the past of kinase activity $\{a\},\sigma_{s|a}^{2}$ (SI). Furthermore, previous measurements (and measurements below) show that $\tau_{1}\ll \tau_{v}$ (17,64,65) and $\tau_{2}\approx \tau_{n}$ (17). Thus, in shallow gradients, we find that the information rate encoded in kinase activity is:

(4) $${\dot{I}}_{s\to a}^{*}\approx \frac{1}{\tau_{v}}\frac{1}{4}\gamma_{a}\frac{\gamma_{r}/\gamma_{a}}{{\left(1\;+\;\sqrt{\gamma_{r}/\gamma_{a}}\right)}^{2}}.$$

Here, we define the dimensionless kinase signal-to-noise ratio $\gamma_{a}=\frac{G_{r}^{2}}{D_{n}}\;r_{0}^{2}\;g^{2}\;\sigma_{v}^{2}\;\tau_{v}$. Eqn. 4 is valid when $\gamma_{a}\ll 1$, which sets the small-signal regime for ${\dot{I}}_{s\to a}^{*}$. We also provide a full expression for ${\dot{I}}_{s\to a}^{*}$ in the SI (SI Eqn. 99). An ideal sensor with no internal noise corresponds to $\gamma_{a}\to \infty$. Taking this limit in Eqn. 4 results in the expression for ${\dot{I}}_{s\to r}^{*}$ in Eqn. 2. Conversely, internal noise degrades information about the signal, and the information rate becomes ${\dot{I}}_{s\to a}^{*}\approx \frac{1}{\tau_{v}}\frac{1}{4}\gamma_{a}$ as $\gamma_{a}\to 0$. The derivation of ${\dot{I}}_{s\to a}^{*}$ also provides the optimal kernel for constructing a running estimate of s(t) from past kinase activity {a}, which we discuss in the SI.

### Single-cell measurements constrain signal and kinase properties

To quantify the information rates above, we then performed single-cell tracking and FRET experiments to measure the parameters characterizing the signal statistics, kinase response function, and kinase noise statistics. As the attractant, we used aspartate (Asp), to which the *E. coli* chemotaxis signaling pathway responds with the highest sensitivity among known attractants (66).

**Figure 2:**
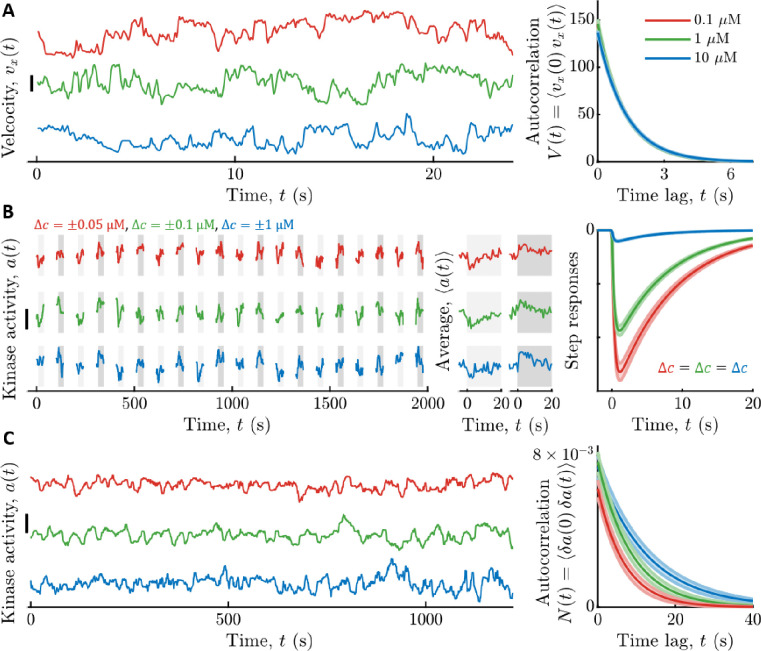
Measured signal statistics and kinase responses and fluctuations in different background ligand concentrations. **A)** Signal statistics. Left: Representative time series of up-gradient velocity $v_{x}$ from three individual cells are shown, one in each aspartate (Asp) concentration $c_{0}$. Scale bar is 20 μm/s. Cells were binned by the fraction of time spent running, $P_{run}$, and the velocity autocorrelation function V(t) was computed by averaging over cells with the median $P_{run}$. The parameters of V(t) were extracted by fitting a decaying exponential to the data. Right: V(t) model fits for each $c_{0}$. The curves are on top of each other. Vertical axis units are (μm/s)^2^. Throughout, shading is standard error of the mean (SEM), and line colors indicate $c_{0}$: Red: 0.1 μM; Green: 1 μM; Blue: 10 μM. **B)** Linear responses. Left: Kinase activity was measured by FRET in blocks of 25 seconds, separated by 65 seconds without illumination. In each block, after 5 s, concentration was stepped up (light gray shading) or down (dark gray shading) around $c_{0}$, then maintained for 20 s, then returned to $c_{0}$. Concentration step sizes Δc were different for each $c_{0}$ (shown above the panel). Shown are three representative cells, one from each $c_{0}$. Scale bar is 0.3. Middle: Average responses of the cells in the left panel to steps up (light gray) and steps down (dark gray). Single-cell responses were fit to extract parameters of the response function $K_{r}(t)$. Right: Model fits for kinase responses to a steps size Δc, using population-median parameters. The gain $G_{r}$ decreases with $c_{0}$. **C)** Noise statistics. Left: Fluctuations in kinase activity were measured in constant background concentrations. Representative time series from three cells are shown, one from each $c_{0}$. Scale bar height is 0.3. Parameters of the slow noise autocorrelation function were fit to single-cell traces using Bayesian filtering (SI). Right: Estimated noise autocorrelation functions with population median parameters. Vertical axis units are kinase activity squared.

To quantify the signal statistics, we recorded trajectories of cells swimming in multiple background concentrations of Asp: $c_{0}=0.1,\;1$, and 10 μM (Fig. 2A). Single cells in the clonal population exhibited a range of phenotypes (61,67–75). Therefore, as before (17), we focused on a typical cell in the population. In particular, we binned cells by the fraction of time spent running, $P_{run}$, and computed V(t) among cells with the median $P_{run}$. The parameters $\sigma_{v}^{2}$ and $\tau_{v}$ in each background $c_{0}$ were then estimated by fitting V(t) with a decaying exponential. These parameters depended weakly on $c_{0}$, and their values in $c_{0}=\;1\;\mu M$ were $\sigma_{v}^{2}=\;146\;\pm \;5\;{\left(\mu m/s\right)}^{2}\;$ and $\tau_{v}=\;1.19\;\pm \;0.01\;s$ (see Fig. S1AB for all values).

We measured kinase response functions as before (17), using a microfluidic device in which we can deliver controlled chemical stimuli with high time resolution (~100 ms) (76). Cells immobilized in the device were delivered ten small positive and negative step changes of Asp concentration around multiple backgrounds $c_{0}$ (Fig. 2B). Kinase responses were measured in single cells through FRET (62,63,76–80) between CheZ-mYFP and CheY-mRFP1. Then we fit each cell’s average response to $K_{r}(t)$ above, and computed the population-median parameter values. Since $\tau_{1}$ estimated this way includes the relatively slow dynamics of CheY-CheZ interactions, we used $\tau_{1}=0$ for calculations below, which only slightly overestimates ${\dot{I}}_{s\to a}^{*}$. The adaptation time $\tau_{2}$ depended weakly on $c_{0}$ (in $c_{0}=1\;\mu M,\tau_{2}=7.4\pm 0.3\;s$) (Fig. S1D), but $G_{r}$ varied significantly: for $c_{0}=\{0.1,1,10\}$ μM we measured $G_{r}=\frac{1}{k_{D}}\{3.2\pm 0.1,2.28\pm 0.05,0.251\pm 0.009\}$ (Fig. S1EF).

The dependence of $G_{r}$ on $c_{0}$ was consistent with the Monod-Wyman-Changeux (MWC) model for kinase activity (27,81–83), which captures numerous experimental measurements (76,78–80,84). In particular, $G_{r}=\frac{1}{r_{0}}G\left(c_{0}\right)$, where $G\left(c_{0}\right)\approx G_{\infty }\frac{c_{0}}{c_{0}+K_{i}}$ is the MWC gain, $K_{i}$ is the dissociation constant of two-state receptors for Asp when in their inactive state, and $G_{\infty }$ is a constant (SI). Thus, in the “linear-sensing” regime ($c_{0}\ll K_{i}$), the gain is constant, $G_{r}=G_{\infty }\frac{1}{k_{D}K_{i}}$, and in the “log-sensing” regime ($c_{0}\gg K_{i}$) (85–87), the gain decreases with background, $G_{r}\approx G_{\infty }/r_{0}$. Fitting the measured $G_{r}$ to the MWC model gave $G_{\infty }=3.5\;\pm \;0.1$ and $K_{i}=\;0.81\;\pm \;0.04\;\mu M$.

Finally, we estimated the parameters of slow kinase fluctuations by measuring kinase activity in single cells experiencing constant Asp concentrations $c_{0}$ (Fig. 2C). The diffusivity $D_{n}$ and time scale $\tau_{n}$ of these fluctuations were extracted from each time series using Bayesian filtering (17,88). We then computed the population-median parameter values. Both of these parameters depended weakly on $c_{0}$, and their values in $c_{0}=\;1\;\mu M\;$ were $D_{n}=\;8.1\;\pm \;0.9\;\times \;10^{-4}\;s^{-1}$ and $\tau_{n}=\;8.7\;\pm \;0.9\;s$ (see Fig. S1CD for all values).

### Comparing *E. coli* to the physical limit

**Figure 3:**
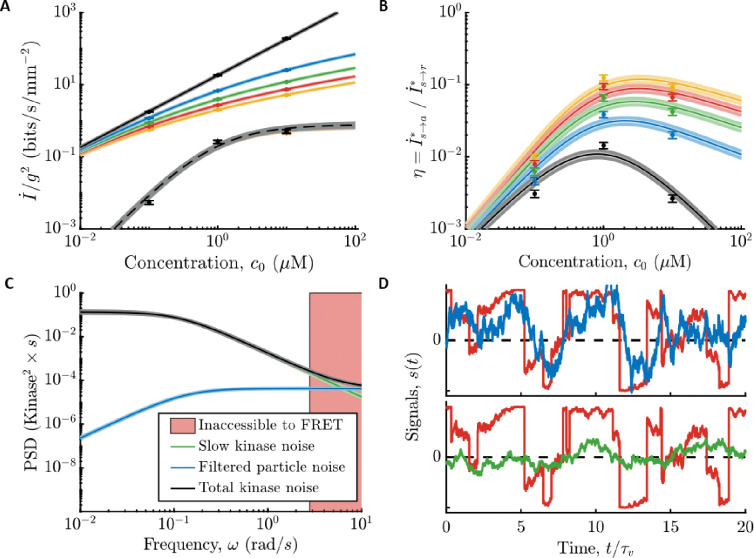
Comparing *E. coli*’s information rates to the physical limit. **A)** Information rates per gradient steepness squared, $g^{2}$, in molecule arrival rate, ${\dot{I}}_{s\to r}^{*}$ (SI Eqn. 60; solid lines), and in kinase activity, ${\dot{I}}_{s\to a}^{*}$ (SI Eqn. 99; dashed lines use the MWC model gain $G\left(c_{0}\right)$ and remaining parameters measured in $c_{0}=1$ μM) for gradients of varying steepness, $g\in \left\{0^{+},\;0.1,\;0.2,\;0.3,\;0.4\right\}{mm}^{-1}$ in black, blue, green, red, yellow, where 0^+^ is the limit of an infinitely shallow gradient. Dots are experimental measurements. Error bars and shading throughout are the SEM. *E. coli* are far from the physical limit when signals are weak and sensor quality matters. **B)** $\eta ={\dot{I}}_{s\to a}^{*}/{\dot{I}}_{s\to r}^{*}$ versus $c_{0}$. Colors and markers are same as in (A). **C)** Fit models for the PSD’s of noise sources, with $c_{0}=1\;\mu M$. Green: Slow noise in kinase activity. Blue: Molecule arrival noise filtered through the kinase response function. Black: Sum of green and blue. Red shading: Experimentally-inaccessible time scales using CheY-CheZ FRET. See also SI Fig. S3 and the Materials and Methods section “Modeling kinase activity.” **D)** Reconstructions of signal, s(t), from time series in Fig. 1. Red: True signal with $c_{0}=1\;\mu M$ and $g\;=\;0.3\;{mm}^{-1}$. Blue: s(t) reconstructed from past r (SI Eqn. 56). Green: s(t) reconstructed from past a (SI Eqn. 94).

Both *E. coli*’s information rate, ${\dot{I}}_{s\to a}^{*}$, and the physical limit, ${\dot{I}}_{s\to r}^{*}$, are proportional to $g^{2}$ in shallow gradients. Therefore, using the measured parameters, we plotted the information rates per $g^{2}$ as functions of $c_{0}$ (Fig. 3A), for values of g in which we previously measured *E. coli*’s gradient-climbing speeds (17). Doing so reveals that *E. coli* are surprisingly far from the physical limit: in shallow gradients, ${\dot{I}}_{s\to a}^{*}$ is at least two orders of magnitude below ${\dot{I}}_{s\to r}^{*}$ across all background concentrations.

To quantify this comparison, we computed the ratio of *E. coli*’s information rate and the physical limit, $\eta \equiv {\dot{I}}_{s\to a}^{*}/{\dot{I}}_{s\to r}^{*}$ (Fig. 3B). In vanishingly small gradients (black curve), η is independent of g. In this regime, ${\dot{I}}_{s\to r}^{*}\propto c_{0}$ in all background concentrations, and the shape of η is determined by the gain of kinase response, $G_{r}$. When $c_{0}\ll K_{i}$, the gain is constant, and η increases with background, $\eta \propto c_{0}$. When $c_{0}\gg K_{i},G_{r}$ decreases and cancels out increasing $c_{0}$, so $\eta \propto 1/c_{0}$. These two regimes are separated by a peak at $c_{0}=K_{i}$, where η≈0.014±0.002at our closest measurement. As the gradient gets steeper, η increases, up to η≈0.1 when $g=\;0.4\;{mm}^{-1}$. This larger value of η does not imply that *E. coli* count nearly every molecule in steeper gradients. Instead, the physical limit saturates (solid lines decreasing with g in Fig. 3A). Thus, in a steep gradient, even a poor sensor can infer the signal with decent accuracy.

In Fig. 3C, we show the power spectral density (PSD) of slow noise in kinase activity (green line) compared to the PSD of filtered molecule arrival noise (blue line) in $c_{0}=\;1\;\mu M$. If *E. coli* were close to the physical limit, nearly all noise in kinase activity would come from filtered molecule arrivals. Instead, slow kinase fluctuations are much larger over the range of frequencies observable in the experiment (Fig. 3C, outside the pink region).

In Fig. 3D, we show the optimal reconstructions of s(t) (Fig. 1), both from past molecule arrival rate {r} and from past kinase activity {a}. The reconstruction from kinase activity is visibly worse, consistent with the much lower information about the signal encoded in the kinase activity. Thus, *E. coli*’s chemosensing is limited by constraints on its internal signal processing, rather than the external physics of ligand diffusion.

## Discussion

Previous work anticipated that *E. coli* would be much closer to the physical limit. Berg and Purcell argued that the change in concentration over a single run in a typical gradient, Δc, could in principle be estimated with uncertainty less than Δc (4). From this, they concluded that the bacterial chemotaxis machinery is nearly optimal. However, their calculation does not imply that bacteria actually achieve that level of accuracy. Ref. (43) fit agent-based simulations to experimental measurements of *Vibrio ordalii* climbing dynamic chemical gradients and argued that this bacterium is within a factor of ~6 of the physical limit. That analysis assumed that cells infer s(t) in independent time windows of duration T=0.1s. However, the signal is correlated over a time $\tau_{v}>T$, and real cells continuously monitor molecule arrivals, allowing them to average out molecule arrival noise for times up to $\tau_{v}$. This increases the theoretical maximum precision, and thus *V. ordalii*’s distance from the limit, by a factor of ${\left(\tau_{v}/T\right)}^{3}={\left(\frac{0.45\;s}{0.1\;s}\right)}^{3}∼90$, due to the $T^{3}$ in the uncertainty about signal ((33) and $\gamma_{r}$ in Eqn. 2). This suggests that chemosensing in other bacterial species is also internally-limited.

Why are *E. coli* so far from the physical limit? One possibility is the physical implementation of their sensory system may impose trade-offs or constraints. For example, the need to operate over a wide range of background concentrations (85–87) suppresses gain in high backgrounds, while the noise stays constant, reducing information. Cells may need to amplify signals above downstream noise sources, requiring the densely-packed arrays seen universally across bacterial species (89), but these might also introduce noise. Indeed, the dense localization of receptors suggests that molecule counting is not limiting, since the optimal strategy in that case would be to uniformly distribute the receptors (4). They also need to sense amino acids, sugars, and peptides (66,90) with different receptors, but the presence of multiple receptor types in the array reduces the response to any one ligand (80). Another possibility is that *E. coli* may be, and likely are, under selection pressures to perform other tasks, such as localize at concentration peaks (91–94). Laboratory strains have been selected for chemotaxis via collective migration assays (95–97). The steep gradients generated during migration, ~1 mm^−1^ or steeper (98–100), might obviate the need for a high-fidelity sensor. Lastly, increasing information about signals might be possible, but too costly in resources or energy to be worth the gain in fitness (18–24,101,102). The mechanism of amplification is not well understood, but recent work has argued that it consumes energy (103–105). These possibilities might be distinguished by measuring information rates of single cells in an isogenic population or information rates of mutants. If any of these cells get closer to the physical limit, it would mean that *E. coli* are not limited by hard implementation constraints, but rather by costs or competing objectives.

Although we found that *E. coli* chemosensing is internally limited, this was only possible because we derived a physical limit that provided a reference point to compare against. While this highlights the value of normative theories, our results also motivate taking seriously the system-specific, internal constraints that may be needed to understand the design of biological systems.

## Supplementary Material

1

## Data Availability

Source data for the main text figures will be provided online with the manuscript. Source data for the Supplementary Figures are contained in a Supplementary Data file.
