# Supplementary material for: E. coli do not count single molecules
Source: ArXiv. 2024 Jul 9:arXiv:2407.07264v1. Preprint. [Version 1] (PMC11261978)
Supplement: Supplementary file 1 [file NIHPP2407.07264v1-supplement-1.pdf]

Supplementary Information for: *E. coli* do not count single molecules

Henry H. Mattingly†,[1], Keita Kamino†,[2], Jude Ong‡,[3], Rafaela Kottou‡,[3], Thierry Emonet*,[3,4,5], Benjamin B. Machta*,[4,5]

[1] Center for Computational Biology, Flatiron Institute
[2] Institute of Molecular Biology, Academia Sinica
[3] Molecular, Cellular, and Developmental Biology, [4] Physics, and [5] QBio Institute, Yale University

† These authors contributed equally.

‡ These authors contributed equally.

* Correspondence to: Benjamin.machta@yale.edu; Thierry.emonet@yale.edu.

Contents

Supplementary figures 1
Background: Drift speed and information rate 3
Equivalence between transfer entropy and predictive information rates 4
Derivation of the information in particle arrivals 5
Optimal kernel for estimating signal from particle arrivals 11
Modeling kinase activity 13
Derivation of the behaviorally-relevant information rate in kinase activity 14
Optimal kernel for estimating signal from kinase activity 19
Information about current versus past signals encoded in kinase activity 20
Appendix A: Causal Wiener filter derivation 22
Supplemental References 25

Supplementary figures

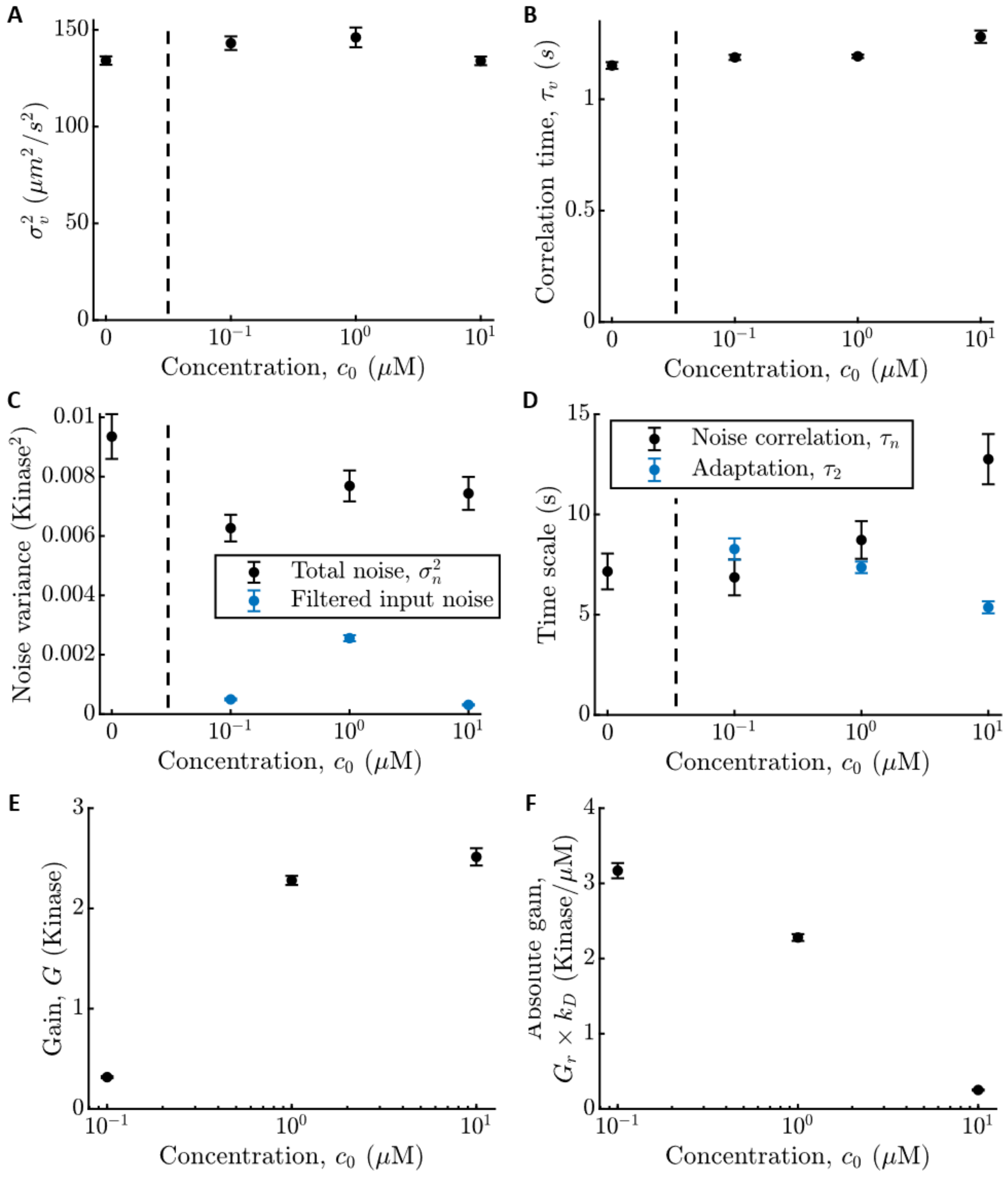

Fig. S1: Measured signal, response, and noise parameter values in different background concentrations. A) Estimated variance of up-gradient velocity, σ_v^2, as a function of background c_0, which together with the gradient steepness g sets the signal strength. Horizontal axes are on log-scale, and vertical dashed lines throughout separate parameters measured at $c_0 = 0$ from those measured at finite c_0. Error bars

throughout are standard error of the mean (see Methods in the main text). **B)** Correlation time of up-gradient velocity, τ_v, which sets the signal correlation time. Parameters in (A) and (B) are those of the median phenotype in Fig. S2, with tumble bias $TB \approx 0.09$. **C)** Variance of the total noise in kinase activity, σ_n^2 (black), and the estimated variance of particle arrival noise filtered through the kinase response kernel (blue) with $\tau_1 = 1/60\ s$ (1,2). **D)** Kinase noise correlation time, τ_n, and kinase response adaptation time, τ_2 (blue). **E)** Gain of kinase response to signal or log-concentration, G. **D)** Gain of kinase response to absolute concentration, $G_c = k_D\ G_r = G/c_0$, where G_r is the gain of kinase responses to particle arrival rate.

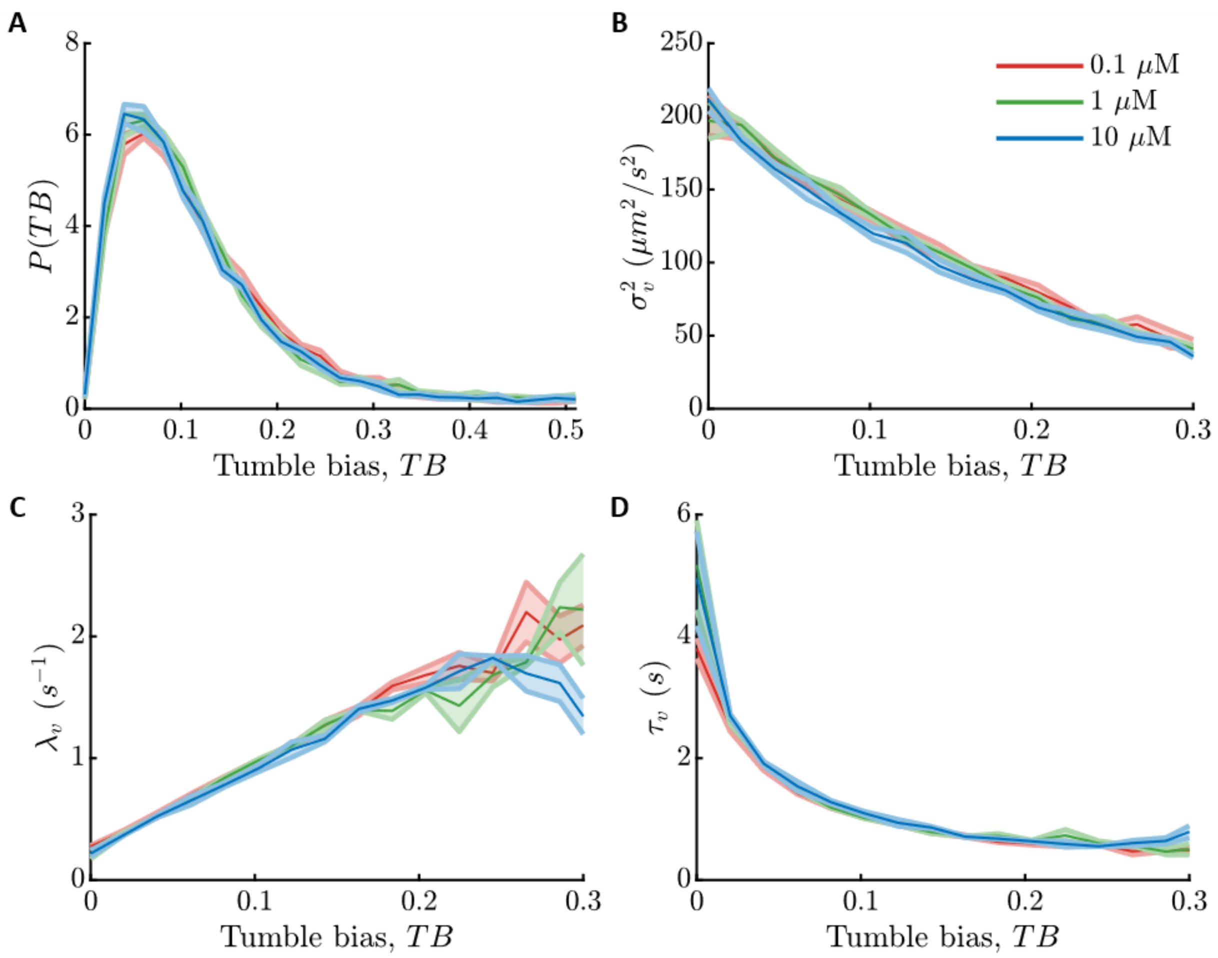

Fig. S2: Swimming parameters as a function of tumble bias in different background concentrations. A) Distribution of tumble bias, $TB = 1 - P_{run}$, or fraction of time cells spend in the tumble state, among cells in an isogenic population. Throughout: red is $c_0 = 0.1$ μM, green is $c_0 = 1$ μM, and blue is $c_0 = 10$ μM. Shading is standard error of the mean (Methods). **B)** Variance of up-gradient velocity, σ_v^2, versus tumble bias, TB. **C)** Velocity decorrelation rate, $\lambda_v = \tau_v^{-1} \approx (1 - \alpha)\ \lambda_{R0} + 2\ D_r$, versus TB. α quantifies how correlated heading is before and after a tumble; λ_{R0} is the average tumble rate; and D_r is the rotational diffusion coefficient (3). **D)** Velocity correlation time, τ_v.

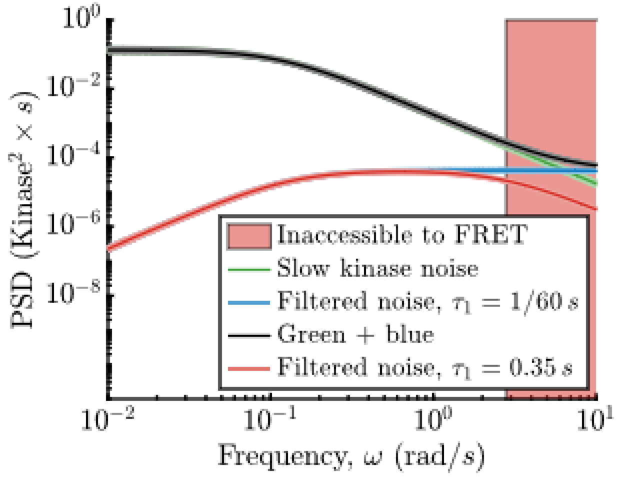

Fig. S3: Noise power spectra. In frequency space, kinase responses to particle arrivals implies that the noise in kinase activity must be larger than filtered particle arrival noise (blue, using $\tau_1 = 1/60\ s$ from biochemistry studies Refs. (1,2)). At low frequencies where we can measure noise and responses with our FRET system (green), this bound is far from saturated. Naively extrapolating to higher frequencies (red shaded region, marked by the value of $1/\tau_1$ measured in FRET experiments) violates this bound (the green line goes below the blue line). This implies either additional noise at high frequencies that is not captured by a single exponential (black line is slow noise, green, plus filtered particle noise, blue) or a slower kinase response time τ_1 (red line is filtered particle noise with $\tau_1 \approx 0.35\ s$ measured in FRET experiments), which could be a necessary by product of the coupling between kinases that creates large gain (thus raising the red line) but also slows down the response. The behaviorally-relevant information rates computed in the main text are relatively insensitive to these choices.

Background: Drift speed and information rate

We recently demonstrated that a cell's drift speed v_d is set by the transfer entropy rate, $\dot{I}^*_{s\to m}$, from current signal $s(t) = \frac{1}{c_0}\frac{dc}{dt}$ to (the trajectory of) swimming behavior $m(t)$ (3). The transfer entropy rate from current signal to swimming behavior is defined as:

$$\dot{I}^*_{s\to m} = \lim_{dt\to 0}\frac{1}{dt} I(m(t+dt); s(t)|\{m(t)\}) \quad (1)$$

$$= \lim_{dt\to 0}\frac{1}{dt}\left\langle \log\left(\frac{P(m(t+dt)|s(t),\{m(t)\})}{P(m(t+dt)|\{m(t)\})}\right)\right\rangle. \quad (2)$$

Here, curly brackets denote the entire past of a variable, up to and including time t. Angled brackets indicate an averaged over the joint distribution of $s(t)$, past $m(t)$, and $m(t+dt)$. This quantifies how strongly the swimming transition probabilities depend on the current signal.

The transfer entropy rate from current signal determines the cell's drift speed (3):

$$\frac{v_d}{v_0} = \frac{(1-\alpha)\,\lambda_{R0}}{(1-\alpha)\,\lambda_{R0} + 2\,D_r}\left(\frac{2}{3}\frac{\dot{I}^*_{s\to m}}{\lambda_{R0}}P_{run}\right)^{1/2} \tag{3}$$

where v_0 is the cell's swimming speed, λ_{R0} is the cell's average tumble rate, α is the persistence of the cell's orientation upon tumbling, D_r is the rotational diffusion coefficient, and P_{run} is the fraction of time the cell spends in the run state.

This transfer entropy obeys a series of data processing inequalities (4,5) because of the feed-forward relationship between molecule arrival rate $r(t)$, kinase activity $a(t)$, and swimming behavior $m(t)$:

$$\dot{I}^*_{s\to r} \geq \dot{I}^*_{s\to a} \geq \dot{I}^*_{s\to m}. \tag{4}$$

Thus, information about current signal available in particle arrivals sets a fundamental upper limit on a cell's gradient climbing speed. Because these information rates set the cell's chemotaxis performance, defined as v_d/v_0, these transfer entropy rates quantify behaviorally-relevant information.

Equivalence between transfer entropy and predictive information rates

Here we demonstrate that the transfer entropy rates above are equivalent to a predictive information rate, under some assumptions that are satisfied by bacterial chemotaxis. This relationship is useful because it allows us to derive expressions for the behaviorally-relevant information rates above.

Below, we will write transfer entropy rate from a signal $s(t)$ to a stochastic process $x(t)$, such as $r(t)$, $a(t)$, or $m(t)$. Starting from the definition above:

$$\dot{I}^*_{s\to x} = \lim_{dt\to 0}\frac{1}{dt} I(x(t+dt); s(t)|\{x(t)\})\,, \tag{5}$$

conditional mutual information can always be written as a difference between two unconditioned mutual information terms:

$$= \lim_{dt\to 0}\frac{1}{dt}\Big(I\big(\{x(t+dt)\}; s(t)\big) - I\big(\{x(t)\}; s(t)\big)\Big)\,. \tag{6}$$

This can be written as

$$= \big[\partial_T I\big(\{x(T)\}; s(t)\big)\big]_{T=t}. \tag{7}$$

Changing variables from T to τ, where $T = t + \tau$, we get:

$$= \big[\partial_\tau I\big(\{x(t+\tau)\}; s(t)\big)\big]_{\tau=0}. \tag{8}$$

Next, we use time stationarity to shift time t by $-\tau$:

$$= \big[\partial_\tau I\big(\{x(t)\}; s(t-\tau)\big)\big]_{\tau=0}. \tag{9}$$

Finally, we can change variables to $\tau \to -\tau$, giving:

$$= -\big[\partial_\tau I\big(\{x(t)\}; s(t+\tau)\big)\big]_{\tau=0}. \tag{10}$$

This last step would not be allowed if the mutual information inside the time derivative was the entire past of s, i.e. $\{s(t+\tau)\}$.

Inside the time derivative above is the "predictive information" (6–8) between the entire past of the stochastic process $x(t)$ up to time t and the signal $s(t)$ at some time τ into the future (if $\tau > 0$). The time derivative of this mutual information or predictive information is a monotonically decreasing function of τ: the value of the signal s at a time further in the future (larger τ) becomes less correlated with past observations and thus harder to predict.

Derivation of the information in particle arrivals

In this section we derive the information rate from current signal $s(t)$ to past particle counts r, which sets a fundamental upper limit on the information rate achievable by a cell. This information rate is given by the following transfer entropy rate:

$$\dot{I}^*_{s\to r} = \lim_{dt\to 0} \frac{1}{dt} I(r(t+dt); s(t)|\{r(t)\})$$
$$= -\left[\partial_\tau I\big(\{r(t)\}; s(t+\tau)\big)\right]_{\tau=0}. \quad (11)$$

Here, $s(t) = \frac{d}{dt}\log(c)$ is the relative rate of change of ligand concentration along the cell's trajectory, and $r(t)$ is the number of ligand molecules per time that arrive at the cell's surface.

The key quantity we need to derive is the mutual information inside of the derivative:

$$I\big(\{r(t)\}; s(t+\tau)\big) = \left\langle \log\left(\frac{P(s(t+\tau)|\{r(t)\})}{P\big(s(t+\tau)\big)}\right)\right\rangle = \left\langle \log\left(\frac{P(\{r(t)\} \mid s(t+\tau))}{P(\{r(t)\})}\right)\right\rangle. \quad (12)$$

In general, it is difficult to derive the conditional distributions above. However, we can make a few simplifying assumptions. First, although the distribution of particle arrival rate $P\big(\{r(t)\} \mid s(t+\tau)\big)$ has Poisson statistics, if a sufficient number of particles arrive at the cell's receptor array per unit time, the Poisson statistics are approximately Gaussian. This approximation is accurate when the cell sees much more than 1 particle per run on average.

Even with this approximation, $P(\{r(t)\}) = \int P\big(\{r(t)\} \mid s(t+\tau)\big)\, P\big(s(t+\tau)\big)\, ds$ is technically not Gaussian because $P\big(s(t+\tau)\big)$ is not Gaussian. However, in shallow gradients (small s), the (roughly) Gaussian particle arrival noise described by $P\big(\{r(t)\} \mid s(t+\tau)\big)$ blurs the structure in $P\big(s(t+\tau)\big)$, making $P(\{r(t)\})$ nearly Gaussian, as well. As a result, we can approximate the mutual information in Eqn. 12 by approximating all distributions as Gaussian, as shown rigorously by others (9–11).

We focus on computing a Gaussian approximation of $P(s(t+\tau)|\{r(t)\})$. For this, we only need to compute the mean $\mu_{s|r}(\tau)$ and variance $\sigma^2_{s|r}(\tau)$. The mutual information can then be computed from:

$$I\big(\{r(t)\}; s(t+\tau)\big) \approx \frac{1}{2}\log\left(\frac{\sigma_s^2}{\sigma^2_{s|r}(\tau)}\right) = -\frac{1}{2}\log\big(1-\rho^2_{rs}(\tau)\big), \quad (13)$$

and the information rate is:

$$\dot{I}^*_{s\to r} = \frac{1}{2}\left[\frac{-\partial_\tau \rho^2_{rs}(\tau)}{1-\rho^2_{rs}(\tau)}\right]_{\tau=0}. \tag{14}$$

Here, σ_s^2 is the marginal variance of $s(t+\tau)$ or $s(t)$, i.e. the variance of the distribution $P\big(s(t+\tau)\big) = P\big(s(t)\big)$ (by time-translation invariance). Then, $\rho^2_{rs}(\tau) = 1 - \frac{\sigma^2_{s|r}(\tau)}{\sigma_s^2}$ is a generalized correlation coefficient between $s(t+\tau)$ and past r, or the fraction reduction of variance in $s(t+\tau)$ upon observing past r.

To determine $\dot{I}^*_{s\to r}$, we now need to calculate the generalized correlation coefficient $\rho^2_{rs}(\tau)$ using models for the dynamics of s and r. Consider a single cell navigating a shallow, static chemical gradient, $c(x) = c_0\, e^{g\,x} \sim c_0(1+g\,x)$, that varies along one spatial dimension, x, in 3D space. In a static gradient, the signal is determined by the cell's motion in the gradient: $s(t) = \frac{d}{dt}\log(c) \approx \frac{1}{c_0}\frac{dc}{dt} = g\, v_x(t)$. As done before (3), we model the cell's up-gradient velocity, and thus the signal, as a Gaussian process with correlation function:

$$\langle s(t)\, s(t')\rangle = g^2\, V(t) = g^2\, \sigma_v^2 \exp\left(-\frac{|t-t'|}{\tau_v}\right). \tag{15}$$

Here, $\sigma_v^2 \approx \frac{v_0^2}{3} P_{run}$ is the variance of the cell's up-gradient velocity, v_0 is its swimming speed, and P_{run} is the fraction of time it spends in the run state; and τ_v is the correlation time of the cell's velocity and the signal, $\tau_v^{-1} = (1-\alpha)\,\lambda_{R0} + 2\,D_r$, where λ_{R0} is the cell's baseline tumble rate, α is the directional persistence, and D_r is the rotational diffusion coefficient.

Then, concentration and particle arrival rate can be modeled as:

$$\frac{dc}{dt} = c_0\, s(t) \tag{16}$$

$$r(t) = k_D\, c(t) + \sqrt{r_0}\,\xi(t). \tag{17}$$

$k_D = 4\,D\,l$ is the diffusion-limited rate constant of particle arrivals to a membrane patch of radius l and for ligand diffusion coefficient D (12–14). The particle arrival noise obeys $\langle \xi(t)\,\xi(t')\rangle = \delta(t-t')$, and $r_0 = k_D\, c_0$ is the particle arrival rate in background concentration c_0.

Since all distributions are approximately Gaussian, the posterior distribution of $s(t+\tau)$ given past $r(t)$ is Gaussian as well: $p(s(t+\tau)|\{r(t)\}) = \mathcal{N}\left(\mu_{s|r}(\tau), \sigma^2_{s|r}(\tau)\right)$. The mean of this distribution, $\mu_{s|r}(\tau)$, can be computed using the causal Wiener filter, $M_r(T)$, which minimizes the following mean squared error $\langle e^2(\tau)\rangle$:

$$\langle e^2(\tau)\rangle = \left\langle\left(s(t+\tau) - \int_{-\infty}^{t} M_r(t-t')\, r(t')\, dt'\right)^2\right\rangle \tag{18}$$

Once the optimal kernel $M_r(T)$ is obtained, the mean of the posterior is $\mu_{s|r}(\tau) = \int_{-\infty}^{t} M_r(t-t')\, r(t')\, dt'$ and the variance is $\sigma^2_{s|r}(\tau) = \langle e^2(\tau)\rangle$. Therefore, to derive the mutual information $I\big(\{r(t)\}; s(t+\tau)\big)$, and thus the information rate $\dot{I}^*_{s\to r}$, we need to derive this Wiener filter. The main challenge in deriving $M_r(T)$ is that it must satisfy the constraint that it is causal: that is, we require that

$M_r(T) = 0$ for $T < 0$. In Appendix A, we derive the necessary equations and explain where they come from, but here we will just apply them to get $M_r(t)$. See also references (8,15,16).

The optimal kernel can be expressed in Fourier space terms of the power spectra of the signal $s(t)$ and the particle arrival rate $r(t)$ as (Appendix A):

$$M_r(\omega) = \frac{1}{\phi_r(\omega)} \left[\frac{S_{rs}(\omega)}{\phi_r^*(\omega)} e^{-i\,\omega\,\tau} \right]^+ . \qquad (19)$$

$M_r(\omega)$ is the Fourier transform of $M_r(T)$, with the Fourier transform defined as $F[f(t)] = \int_{-\infty}^{\infty} f(t)\, e^{i\,\omega\,t}\, dt$ and inverse transform defined as $F^{-1}[f(\omega)] = \frac{1}{2\pi}\int_{-\infty}^{\infty} f(\omega)\, e^{-i\,\omega\,t}\, d\omega$. $\phi_r(\omega)$ is the causal part of the spectral decomposition of $S_r(\omega)$ (defined below and in Appendix A), where $S_r(\omega)$ is the power spectrum of r. $\phi_r^*(\omega)$ is its (anti-causal) complex conjugate. $S_{rs}(\omega)$ is the cross-spectra of r and s, equivalent to the Fourier transform of $C_{rs}(\tau)$, where $C_{rs}(\tau) = \langle (r(t) - r_0) s(t+\tau) \rangle$ is the cross-correlation of s and r in the time domain. Finally, $[f(\omega)]^+$ indicates the causal part of the inverse Fourier transform of $f(\omega)$, which can be found by taking the inverse Fourier transform of $f(\omega)$, multiplying the result by a Heaviside step function in the time domain, and then taking the Fourier transform.

To derive these various quantities, we take the Fourier transforms of Eqns. 16 and 17, and then solve for the Fourier transforms of our variables $c(\omega)$ and $r(\omega)$:

$$c(\omega) = \frac{c_0}{\frac{\epsilon}{\tau_v} - i\,\omega} s(\omega) \qquad (20)$$

$$r(\omega) = k_D\, c(\omega) + \sqrt{r_0}\, \xi(\omega). \qquad (21)$$

Here we have introduced a small, dimensionless parameter $\epsilon \ll 1$ that we will take to zero later. Physically, this is as if the cell experiences a weak restoring force back to regions where concentration $c(x) = c_0$. Without it, the correlation function of $c(t)$, which is proportional to the cell's mean squared displacement, would diverge at long times. Everything else remains bounded and well-defined as ϵ goes to zero.

From these and the correlation function of $s(t)$, we derive the following spectra:

$$S_s(\omega) = F[C_s(T)] = \frac{2\, g^2 \frac{\sigma_v^2}{\tau_v}}{\frac{1}{\tau_v^2} + \omega^2} \qquad (22)$$

$$S_r(\omega) = F[C_r(T)] = \frac{r_0^2}{\frac{\epsilon^2}{\tau_v^2} + \omega^2} S_s(\omega) + r_0 \qquad (23)$$

$$S_{rs}(\omega) = S_{sr}^*(\omega) = F[C_{rs}(T)] = \frac{r_0}{\frac{\epsilon}{\tau_v} + i\,\omega} S_s(\omega) \qquad (24)$$

where $C_s(T) = \langle s(t)\, s(t+T) \rangle$, $C_r(T) = \langle (r(t) - r_0)\, (r(t+T) - r_0) \rangle$, and $C_{rs}(T) = \langle (r(t) - r_0)\, s(t + T) \rangle$.

As explained in Appendix A, to find the optimal causal kernel, we need to decompose $S_r(\omega)$ into the product of a causal and an anti-causal part. This requires finding the zeros and poles of $S_r(\omega)$. The zeros satisfy $S_r(\omega = i\, z_r) = 0$, and therefore are the complex solutions to the equation:

$$2\, r_0\, g^2\, \sigma_v^2\, \tau_v^3 + (\epsilon^2 + \tau_v^2\, \omega^2)\, (1 + \tau_v^2\, \omega^2) = 0 \tag{25}$$

or, defining $\gamma_r = 2\, r_0\, g^2\, \sigma_v^2\, \tau_v^3$:

$$\gamma_r + (\epsilon^2 + \tau_v^2\, \omega^2)\, (1 + \tau_v^2\, \omega^2) = 0. \tag{26}$$

γ_r is a dimensionless signal-to-noise ratio parameter, where the signal is $r_0^2\, g^2\, \sigma_v^2\, \tau_v^3$ (the prefactor of the first term in $S_r(\omega)$ when ω is rescaled by $1/\tau_v$) and the noise is r_0 (the second term in $S_r(\omega)$).

The zeros of $S_r(\omega)$ are:

$$i\, z_{r,1} = i \frac{1}{\sqrt{2}\, \tau_v} \sqrt{1 + \epsilon^2 + \sqrt{(1-\epsilon^2)^2 - 4\, \gamma_r}}, \qquad i\, z_{r,2} = i \frac{1}{\sqrt{2}\, \tau_v} \sqrt{1 + \epsilon^2 - \sqrt{(1-\epsilon^2)^2 - 4\, \gamma_r}}, \tag{27}$$

as well as their complex conjugates, z_1^* and z_2^*. As $\epsilon \to 0$, these will simplify to:

$$i\, z_{r,1} = i\, \frac{1}{\sqrt{2}\, \tau_v} \sqrt{1 + \sqrt{1 - 4\, \gamma_r}}, \qquad i\, z_{r,2} = i\, \frac{1}{\sqrt{2}\, \tau_v} \sqrt{1 - \sqrt{1 - 4\, \gamma_r}}, \tag{28}$$

Note that there are several equivalent forms for these zeros, and they change from being fully imaginary to complex when $\gamma_r > 1/4$.

The poles of $S_r(\omega)$ satisfy $\frac{1}{S_r(\omega = i\, p_r)} = 0$ and are given by $i\, p_{r,1} = i \frac{\epsilon}{\tau_v}$ and $i\, p_{r,2} = i \frac{1}{\tau_v}$, as well as their complex conjugates $p_{r,1}^*$ and $p_{r,2}^*$.

Power spectral densities of real, stable, causal systems can generally be decomposed into causal and anti-causal parts ("Wiener-Hopf factorization") (17–19):

$$S_r(\omega) = \phi_r(\omega)\, \phi_r^*(\omega) \tag{29}$$

where

$$\phi_r(\omega) = \sqrt{r_0}\, \frac{(z_{r,1} - i\, \omega)\, (z_{r,2} - i\, \omega)}{(p_{r,1} - i\, \omega)\, (p_{r,2} - i\, \omega)} \tag{30}$$

has zeros and poles with negative imaginary parts, and $\phi_r^*(\omega)$ is its complex conjugate.

Next, we need the causal part of (see Appendix A):

$$\frac{S_{rs}(\omega)}{\phi_r^*(\omega)} e^{-i\,\omega\,\tau} = \frac{\sqrt{r_0}}{\frac{\epsilon}{\tau_v} + i\, \omega} S_s(\omega) \frac{(p_{r,1} + i\, \omega)\, (p_{r,2} + i\, \omega)}{(z_{r,1} + i\, \omega)\, (z_{r,2} + i\, \omega)} e^{-i\,\omega\,\tau} \tag{31}$$

$$= \frac{\sqrt{r_0}}{\frac{\epsilon}{\tau_v} + i\, \omega} \frac{2\, g^2 \frac{\sigma_v^2}{\tau_v}}{\frac{1}{\tau_v^2} + \omega^2} \frac{\left(\frac{\epsilon}{\tau_v} + i\, \omega\right) \left(\frac{1}{\tau_v} + i\, \omega\right)}{(z_{r,1} + i\, \omega)\, (z_{r,2} + i\, \omega)} e^{-i\,\omega\,\tau} \tag{32}$$

$$= \sqrt{r_0}\, \frac{2\, g^2 \frac{\sigma_v^2}{\tau_v}}{\left(\frac{1}{\tau_v} - i\,\omega\right)} \frac{1}{\left(z_{r,1} + i\,\omega\right)\left(z_{r,2} + i\,\omega\right)} e^{-i\,\omega\,\tau} \tag{33}$$

$$= \frac{\gamma_r}{\sqrt{r_0}\, \tau_v^4} \frac{1}{\left(\frac{1}{\tau_v} - i\,\omega\right)} \frac{1}{\left(z_{r,1} + i\,\omega\right)\left(z_{r,2} + i\,\omega\right)} e^{-i\,\omega\,\tau} \tag{34}$$

One approach would be to compute the inverse Fourier transform of $\frac{S_{rs}(\omega)}{\phi_r^*(\omega)}$, apply the time shift forward by τ implied by $e^{-i\,\omega\,\tau}$, multiply the result by a Heaviside step function $\Theta(T)$, and compute the Fourier transform of the result. An alternative approach is to compute the partial fraction decomposition of the expression above and keep only the terms with poles and zeros that have negative imaginary part:

$$\frac{S_{rs}(\omega)}{\phi_r^*(\omega)} e^{-i\,\omega\,\tau} = \frac{A}{\left(\frac{1}{\tau_v} - i\,\omega\right)} + \frac{B}{\left(z_{r,1} + i\,\omega\right)} + \frac{C}{\left(z_{r,2} + i\,\omega\right)} \tag{35}$$

for unknown A, B, and C. Only the pole of the first term ($\omega = -i\frac{1}{\tau_v}$) has negative imaginary part, so we only need to compute A to get the causal part of this expression. With some algebra, this is:

$$A = \left[\frac{\gamma_r}{\sqrt{r_0}\, \tau_v^4} \frac{1}{\left(z_{r,1} + i\,\omega\right)\left(z_{r,2} + i\,\omega\right)} e^{-i\,\omega\,\tau}\right]_{\omega = -i\frac{1}{\tau_v}} \tag{36}$$

$$= \frac{\gamma_r}{\sqrt{r_0}\, \tau_v^2} \frac{1}{\left(1 + \tau_v\, z_{r,1}\right)\left(1 + \tau_v\, z_{r,2}\right)} e^{-\frac{\tau}{\tau_v}}, \tag{37}$$

and the causal part of $\frac{S_{rs}(\omega)}{\phi_r^*(\omega)} e^{-i\,\omega\,\tau}$ is then:

$$\left[\frac{S_{rs}(\omega)}{\phi_r^*(\omega)} e^{-i\,\omega\,\tau}\right]^+ = \frac{\gamma_r}{\sqrt{r_0}\, \tau_v^2} \frac{1}{\left(1 + \tau_v\, z_{r,1}\right)\left(1 + \tau_v\, z_{r,2}\right)} \frac{1}{\left(\frac{1}{\tau_v} - i\,\omega\right)} e^{-\frac{\tau}{\tau_v}}. \tag{38}$$

Finally, the optimal kernel that computes the mean of $p(s(t+\tau)|\{r(t)\})$, $\mu_{s|r}(\tau)$, is (Appendix A):

$$M_r(\omega) = \frac{1}{\phi_r(\omega)} \left[\frac{S_{rs}(\omega)}{\phi_r^*(\omega)} e^{-i\,\omega\,\tau}\right]^+ \tag{39}$$

$$= e^{-\frac{\tau}{\tau_v}} \frac{\gamma_r}{r_0\, \tau_v^2} \frac{1}{\left(1 + \tau_v\, z_{r,1}\right)\left(1 + \tau_v\, z_{r,2}\right)} \frac{-i\,\omega}{\left(z_{r,1} - i\,\omega\right)\left(z_{r,2} - i\omega\right)}, \tag{40}$$

after taking ϵ to zero. We convert this kernel to the time domain and discuss its properties in the next section.

The variance of $p(s(t+\tau)|\{r(t)\})$, $\sigma_{s|r}^2(\tau)$, is (Appendix A, Eqn. 127):

$$\sigma_{s|r}^2(\tau) = \sigma_s^2 - \frac{1}{2\pi} \int_{-\infty}^{\infty} S_{rs}^*(\omega)\, e^{i\,\omega\,\tau}\, M_r(\omega)\, d\omega \tag{41}$$

$$= \sigma_s^2 \left(1 - e^{-2\frac{\tau}{\tau_v}} \frac{\gamma_r}{\left(1 + \tau_v\, z_{r,1}\right)^2 \left(1 + \tau_v\, z_{r,2}\right)^2} \right) \tag{42}$$

where we used $\sigma_s^2 = g^2 \sigma_v^2 = \gamma/(2\, r_0\, \tau_v^3)$. Then the correlation coefficient $\rho_{rs}^2(\tau)$ is:

$$\rho_{rs}^2(\tau) = 1 - \frac{\sigma_{s|r}^2(\tau)}{\sigma_s^2} = e^{-2\frac{\tau}{\tau_v}} \frac{\gamma_r}{\left(1 + \tau_v\, z_{r,1}\right)^2 \left(1 + \tau_v\, z_{r,2}\right)^2}. \tag{43}$$

Finally, using Eqn. 14 from above, we find that the behaviorally-relevant information available in particle counts is:

$$\dot{I}_{s \to r}^* = \frac{1}{\tau_v} \frac{\rho_{rs}^2(\tau = 0)}{1 - \rho_{rs}^2(\tau = 0)} = \frac{1}{\tau_v} \frac{\gamma_r \frac{1}{\left(1 + \frac{1}{\sqrt{2}} \sqrt{1 + \sqrt{1 - 4\,\gamma_r}}\right)^2 \left(1 + \frac{1}{\sqrt{2}} \sqrt{1 - \sqrt{1 - 4\,\gamma_r}}\right)^2}}{1 - \gamma_r \frac{1}{\left(1 + \frac{1}{\sqrt{2}} \sqrt{1 + \sqrt{1 - 4\,\gamma_r}}\right)^2 \left(1 + \frac{1}{\sqrt{2}} \sqrt{1 - \sqrt{1 - 4\,\gamma_r}}\right)^2}}. \tag{44}$$

Expanding around small SNR γ gives:

$$\dot{I}_{s \to r}^* \approx \frac{1}{\tau_v} \frac{\gamma_r}{4} = \frac{1}{2} r_0\, g^2\, \sigma_v^2\, \tau_v^2. \tag{45}$$

Note that for small signals, Eqn. 44 can be written $\dot{I}_{s \to r}^* \approx \frac{1}{\tau_v} \rho_{rs}^2(\tau = 0) \approx \frac{2}{\tau_v} I\big(\{r(t)\}; s(t)\big)$.

The optimal kernel in the time domain and the information rate remain real when $\gamma_r > 1/4$, even though $z_{r,1}$ and $z_{r,2}$ become complex. In this regime, they can be written:

$$z_{r,1} = \frac{1}{2\,\tau_v} \left(\sqrt{2\sqrt{\gamma_r} + 1} - i \sqrt{2\sqrt{\gamma_r} - 1} \right), \qquad z_{r,2} = \frac{1}{2\,\tau_v} \left(\sqrt{2\sqrt{\gamma_r} + 1} + i \sqrt{2\sqrt{\gamma_r} - 1} \right). \tag{46}$$

The optimal kernel in frequency space can be written:

$$M_r(\omega) = e^{-\frac{\tau}{\tau_v}} \frac{\gamma_r}{r_0\, \tau_v^2} \frac{1}{\left(1 + \tau_v\, z_{r,1}\right)\left(1 + \tau_v\, z_{r,2}\right)} \frac{-i\,\omega}{\left(z_{r,1} - i\,\omega\right)\left(z_{r,2} - i\omega\right)} \tag{47}$$

$$= e^{-\frac{\tau}{\tau_v}} \frac{\gamma_r}{r_0\, \tau_v^2} \frac{1}{\left(1 + \sqrt{1 + 2\sqrt{\gamma_r}} + \sqrt{\gamma_r}\right)} \frac{-i\,\omega}{\left(z_{r,1} - i\,\omega\right)\left(z_{r,2} - i\omega\right)}, \tag{48}$$

the correlation coefficient at $\tau = 0$ can be written:

$$\rho_{rs}^2(\tau = 0) = \frac{\gamma_r}{\left(1 + z_{r,1}\,\tau_v\right)^2 \left(1 + z_{r,2}\,\tau_v\right)^2} \tag{49}$$

$$= \frac{\gamma_r}{\left|1 + z_{r,1}\,\tau_v\right|^4} \tag{50}$$

$$= \frac{\gamma_r}{\left(1+\sqrt{1+2\sqrt{\gamma_r}}+\sqrt{\gamma_r}\right)^2}, \tag{51}$$

and the information rate is:

$$\dot{I}^*_{s\to r} = \frac{1}{\tau_v} \frac{\frac{\gamma_r}{\left(1+\sqrt{1+2\sqrt{\gamma_r}}+\sqrt{\gamma_r}\right)^2}}{1-\frac{\gamma_r}{\left(1+\sqrt{1+2\sqrt{\gamma_r}}+\sqrt{\gamma_r}\right)^2}}. \tag{52}$$

For small γ_r, this reduces again to Eqn. 45.

Optimal kernel for estimating signal from particle arrivals

To get the time-domain kernel mapping past particle arrival rate $r(t)$ to signal $s(t+\tau)$, $M_r(T)$,we take the inverse Fourier transform of $M_r(\omega)$, defined as $IFT[f(\omega)] = \frac{1}{2\pi}\int_{-\infty}^{\infty} f(\omega)\, e^{-i\,\omega\,t} d\omega$. $M_r(T)$ has the form of a sum of two exponentials, with real exponents when $\gamma_r \leq 1/4$ and complex ones when $\gamma_r > 1/4$. For $\gamma_r < 1/4$, the kernel in the time domain is:

$$M_r(T) = e^{-\frac{\tau}{\tau_v}} \frac{\gamma_r}{r_0\,\tau_v^2} \frac{1}{(1+z_{r,1}\,\tau_v)\,(1+z_{r,2}\,\tau_v)} \frac{\left(z_{r,1}\, e^{-z_{r,1}\,T} - z_{r,2}\, e^{-z_{r,2}\,T}\right)}{z_{r,1}-z_{r,2}}\, \Theta(T), \tag{53}$$

where $\Theta(T)$ is the Heaviside step function, indicating that the kernel is indeed causal.

The optimal kernel $M_r(T)$ essentially computes the time derivative of concentration, while also averaging out shot noise from particle arrivals. It has several notable features. First, it is biphasic and exhibits perfect adaptation, a hallmark of the chemotaxis pathway. Any derivative operation should adapt perfectly because it should only respond to *changes* in the input.

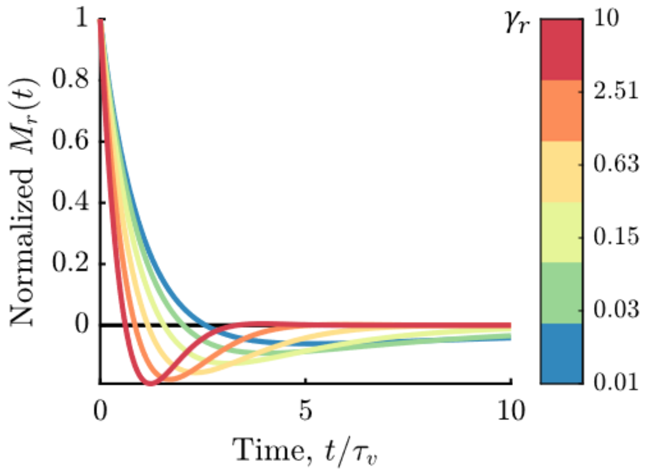

Fig. S4: Optimal kernel for inferring current signal, $s(t)$, from past particle arrivals, r. Colors indicate different values of the signal-to-noise ratio γ_r, marked on the right. Each kernel is normalized so that $M_r(0) = 1$.

It is interesting to examine how the time scales of the optimal kernel are set by the signal-to-noise ratio $\gamma_r = 2\, r_0\, g^2\, \sigma_v^2\, \tau_v^3$. The initial response time scale is set by $z_{r,1}^{-1}$ and its adaptation time scale is set by $z_{r,2}^{-1}$. When the inputs are very noisy, i.e. as $\gamma_r \to 0$, $z_{r,1}^{-1}$ gets longer but saturates at τ_v:

$$z_{r,1}^{-1}(\gamma_r \to 0) \;\approx\; \tau_v \left(1 - \frac{\gamma_r}{2}\right). \tag{54}$$

This makes sense because it maximally averages out shot noise, but only for as long as past signals are correlated with the current signal. As the SNR increases, this initial averaging time gets shorter.

As the inputs get noisier, i.e. as $\gamma_r \to 0$, the adaptation time approaches:

$$z_{r,2}^{-1}(\gamma_r \to 0) \;\approx\; \tau_v \left(\frac{1}{\sqrt{\gamma_r}} - \frac{\sqrt{\gamma_r}}{2}\right). \tag{55}$$

This shows that the adaptation time can become long compared to τ_v when $\gamma_r < 1/4$.

Interestingly, in this regime, the kernel $M_r(T)$ has the same functional form as the phenomenological kernel we measured previously (3) (after transforming the input quantity from $s(t)$ to $c(t)$).

When signal and noise have similar strength $\gamma_r = 1/4$, $z_{r,1} = z_{r,2} = z_r = \frac{1}{\sqrt{2}}\, \tau_v{}^{-1}$, and the optimal kernel becomes:

$$M_r(T) = e^{-\frac{\tau}{\tau_v}} \frac{\gamma_r}{r_0\, \tau_v^2} \frac{1}{\left(1 + \frac{1}{\sqrt{2}}\right)^2} e^{-z_r T} (1 - z_r\, T)\, \Theta(T). \tag{56}$$

When SNR is high $\gamma_r > 1/4$, $z_{r,1}$ and $z_{r,2}$ become complex. However, since they are complex conjugates of each other, the kernel remains real:

$$\begin{aligned} M_r(T) &= e^{-\frac{\tau}{\tau_v}} \frac{\gamma_r}{r_0\, \tau_v^2} \frac{1}{(1 + z_{r,1}\, \tau_v)\,(1 + z_{r,2}\, \tau_v)} e^{-\mathrm{Re}[z_{r,2}]\, T} \left(\cos\!\left(\mathrm{Im}[z_{r,2}]\, T\right) - \frac{\mathrm{Re}[z_{r,2}]}{\mathrm{Im}[z_{r,2}]} \sin\!\left(\mathrm{Im}[z_{r,2}]\, T\right)\right) \Theta(T) \\ &= e^{-\frac{\tau}{\tau_v}} \frac{\gamma_r}{r_0\, \tau_v^2} \frac{1}{\left(1 + \sqrt{1 + 2\sqrt{\gamma_r}} + \sqrt{\gamma_r}\right)} e^{-\frac{1}{2}\sqrt{2\sqrt{\gamma_r}+1}\frac{T}{\tau_v}} \times \\ &\qquad \left(\cos\left(\frac{1}{2}\sqrt{2\sqrt{\gamma_r} - 1}\, \frac{T}{\tau_v}\right) - \sqrt{\frac{2\sqrt{\gamma_r} + 1}{2\sqrt{\gamma_r} - 1}} \sin\left(\frac{1}{2}\sqrt{2\sqrt{\gamma_r} - 1}\, \frac{T}{\tau_v}\right)\right) \Theta(T) \end{aligned} \tag{57}$$

The optimal kernel, $M_r(T)$, is plotted in Fig. S4 for varying values of γ_r.

As the SNR γ_r increases, the initial response time and the adaptation time both get shorter. Although the kernel oscillates, its decay rate is faster than the period of oscillations. The time scales of decay and oscillation are closest to each other, and thus the oscillation amplitude is largest, when γ_r is large: in the limit that $\gamma_r \to \infty$, $\mathrm{Re}[z_{r,2}] = \mathrm{Im}[z_{r,2}] = \gamma_r{}^{1/4}$. Even in this limit, the peak of the kernel following the first

negative lobe occurs at time $T = 3\pi\, \gamma_r^{-1/4}$ and is smaller than the kernel's maximum value ($M_r(T = 0)$) by a factor of $e^{-3\pi/2} \sim 0.009$. Thus, the oscillations are small. $M_r(T)$ transitions continuously between the forms above as γ_r varies.

The results of this and previous section could also be derived using the continuous-time Kalman-Bucy filter (20,21). That approach provides a pair of ODEs for the estimator of s (i.e. conditional mean $\mu_{s|r}$) and its uncertainty (i.e. the conditional variance $\sigma^2_{s|r}$) that are driven by the observations, $r(t)$. Once $\sigma^2_{s|r}$ reaches steady state in that formulation (consistent with our assumption of stationarity here), the ODE for $\mu_{s|r}$ can be solved in terms of a kernel convolved with past $r(t)$, which is identical to the optimal kernel above.

Modeling kinase activity

In shallow gradients, CheA kinases respond approximately linearly to recent signals. We model kinase responses, $a(t)$, to past particle arrival rates, $r(t)$, in background particle arrival rate r_0, as:

$$a(t) = a_0 - \int_{-\infty}^{t} K_r(t - t')\, (r(t') - r_0)\, dt' + \eta(t). \tag{58}$$

The response function to particle arrival rate, $K_r(T)$, is:

$$K_r(t) = G_r \left(\left(\frac{1}{\tau_1} + \frac{1}{\tau_2} \right) \exp\left(-\left(\frac{1}{\tau_1} + \frac{1}{\tau_2} \right) t \right) - \frac{1}{\tau_2} \exp\left(-\frac{t}{\tau_2} \right) \right) \Theta(t), \tag{59}$$

We note that in our previous work (3) we modeled kinase responses to signals, $s(t)$, directly. In the Methods section of the main text, we show how to convert between these representations (Equation 15 in the Methods).

The Fourier transform of this kernel is:

$$K_r(\omega) = \frac{G_r}{\tau_1} \frac{(-i\,\omega)}{\left(\frac{1}{\tau_2} - i\,\omega \right) \left(\frac{1}{\tau_1} + \frac{1}{\tau_2} - i\,\omega \right)}. \tag{60}$$

Particle arrival noise filtered through this kernel has spectrum:

$$N_r(\omega) = r_0\, |K_r(\omega)|^2. \tag{61}$$

In experiments, we measure responses to absolute changes in concentration $c(t)$, with response kernel $K_c(T)$, which has the same form as $K_r(T)$ above, but with gain G_c. Then, we convert G_c to G_r via $G_r = G_c/k_D$, and thus convert $K_c(T)$ to $K_r(T)$. With this, the intensity of filtered particle noise in Eqn. 61 is proportional to $G_r^2\, r_0 = G_c^2\, c_0/k_D$. This conversion implies that *E. coli* respond to every particle arriving at their surface, which is unlikely. Instead, one might use an effective $k_D^{eff} < k_D$ to do the conversion above, which would increase our estimate for the intensity of filtered particle noise, being proportional to $G_c^2\, c_0/k_D^{eff}$. However, modeling the filtered particle noise with $k_D^{eff} = k_D$ maximizes our estimate of *E. coli*'s information rate. Since we find that *E. coli* are far from the physical limit, this is a conservative modeling choice.

Next, we consider modeling noise in kinase activity. As explained in Fig. S3 above, the FRET system we use for measuring kinase activity has limited time resolution, about $0.3\ s$. This allows us to constrain slow fluctuations in kinase activity, whose correlation function is characterized by a single decaying exponential function (3,22):

$$\langle \eta(t)\eta(t')\rangle = N_n(t-t') = \sigma_n^2 \exp\left(-\frac{|t-t'|}{\tau_n}\right) = D_n\, \tau_n \exp\left(-\frac{|t-t'|}{\tau_n}\right). \quad (62)$$

The parameters here are the long-time variance σ_n^2 and the correlation time τ_n, which are related to the diffusivity of the noise by $D_n = \sigma_n^2/\tau_n$. The power spectrum of this noise is

$$N_n(\omega) = \frac{2\, D_n}{\frac{1}{\tau_n^2} + \omega^2}. \quad (63)$$

There can also be noise at higher frequencies that we don't observe. Kinase responses to particle arrival noise set a minimum noise level at all frequencies. At high frequencies, simply extrapolating the power spectrum in Eqn. 63 drops below the implied filtered particle noise in Eqn. 61 if we take that τ_1 in Eqn. 59 to be the value measured previously in biochemical studies (1,2), $\tau_1 \approx 1/60\ s$. One possibility is that cooperativity of the receptor-kinase lattice slows down τ_1 to a value closer to what we measure in FRET, $\tau_1 \approx 0.35\ s$. In this case, extrapolating the slow noise to high frequencies does not cause any problems.

To avoid having unphysical noise power at high frequencies, we take the total noise in kinase activity to be a sum of the measured slow noise in Eqn. 63 plus the filtered particle arrival noise in Eqn. 61. There are likely other noise sources at high frequencies, so this modeling choice maximizes our estimate of *E. coli*'s information rate. Since we find that *E. coli* are far from the physical limit, this is a conservative modeling choice. Ultimately, even if we only model noise in kinase activity as being the slow, measurable noise, the effects on the numerical values of the information rate are small.

Before continuing, we will make an additional simplifying assumption. The adaptation time of kinase responses, τ_2, and the correlation time of kinase noise, τ_n, are each roughly $\sim 10\ s$. Therefore, below we will also assume $\tau_2 \approx \tau_n$, which also has small quantitative effects on the results. These simplifications also allow us to derive interpretable analytical expressions.

Derivation of the behaviorally-relevant information rate in kinase activity

In this section, we derive the information about current signal encoded in the kinase activity of a typical *E. coli* cell. Here, we seek an expression for the following transfer entropy rate:

$$\begin{aligned}\dot{I}^*_{s\to a} &= \lim_{dt\to 0} \frac{1}{dt} I(a(t+dt); s(t)|\{a(t)\}) \\ &= -\big[\partial_\tau I\big(\{a(t)\}; s(t+\tau)\big)\big]_{\tau=0}. \quad (64)\end{aligned}$$

Again, the calculation centers on calculating the mutual information between past kinase activity a and signal at some time τ into the future, $I\big(\{a(t)\}; s(t+\tau)\big)$. The quantity we need to derive this is the posterior distribution of signal given past kinase activity, $P(s(t+\tau)|\{a(t)\})$. Past measurements by us

and others (3,22,23) have shown that kinase activity in wild type cells (i.e. cells with all receptor types and with their adaptation system intact) is well-approximated by a Gaussian process. Because of this, and because we consider shallow gradients, we only need the variance of $P(s(t+\tau)|\{a(t)\})$ to compute the mutual information to leading order in g (see the section **Derivation of the behaviorally-relevant information rate in particle arrivals**, above). Thus, we can approximate s and a as jointly Gaussian distributed.

With the approximation that s and a are also jointly Gaussian distributed, $P(s(t+\tau)|\{a(t)\})$ is Gaussian, and therefore we again need to compute a mean $\mu_{s|a}(\tau)$ and a variance $\sigma^2_{s|a}(\tau)$. Then, the mutual information can then be computed from:

$$I(\{a(t)\}; s(t+\tau)) = \frac{1}{2}\log\left(\frac{\sigma_s^2}{\sigma_{s|a}^2(\tau)}\right) = -\frac{1}{2}\log\left(1-\rho_{as}^2(\tau)\right), \tag{65}$$

and the predictive information rate is

$$\dot{I}^*_{s\to a} = \frac{1}{2}\left[\frac{-\partial_\tau \rho_{as}^2(\tau)}{1-\rho_{as}^2(\tau)}\right]_{\tau=0} \tag{66}$$

Here, $\rho_{as}^2(\tau) = 1 - \frac{\sigma_{s|a}^2(\tau)}{\sigma_s^2}$ is the generalized correlation between $s(t+\tau)$ and past a, or the fraction reduction of variance in $s(t+\tau)$ upon observing past a.

To compute the rate of information transfer from current signal $s(t)$ to kinase activity $a(t)$, we need the conditional mean and variance of $s(t+\tau)$, $\mu_{s|a}(\tau)$ and $\sigma^2_{s|a}(\tau)$. These in turn require deriving the kernel $M_a(T)$ that maps past kinase activity a to the conditional mean, $\mu_{s|a}(\tau)$. This can again be derived using Wiener filtering theory and expressed in terms of the power spectra of s and a. These are:

$$S_s(\omega) = F[C_s(T)] = \frac{2\, g^2 \frac{\sigma_v^2}{\tau_v}}{\frac{1}{\tau_v^2} + \omega^2} \tag{67}$$

$$S_a(\omega) = F[C_a(T)] = |K_r(\omega)|^2\, S_r(\omega) + N_{n(\omega)} \tag{68}$$

$$= |K_r(\omega)|^2 \left(r_0^2 \frac{S_s(\omega)}{\omega^2} + r_0\right) + \frac{2\, D_n}{\frac{1}{\tau_2^2} + \omega^2} \tag{69}$$

$$= \left(\frac{G_r}{\tau_1}\right)^2 \frac{\omega^2}{\left(\frac{1}{\tau_2^2} + \omega^2\right)\left(\left(\frac{1}{\tau_1} + \frac{1}{\tau_2}\right)^2 + \omega^2\right)} \left(r_0^2 \frac{S_s(\omega)}{\omega^2} + r_0\right) + \frac{2\, D_n}{\frac{1}{\tau_2^2} + \omega^2} \tag{70}$$

$$S_{as}(\omega) = S_{sa}^*(\omega) = F[C_{as}(T)] = -K_r^*(\omega)\, S_{rs}(\omega) \tag{71}$$

$$= -\frac{G_r}{\tau_1} \frac{r_0}{\left(\frac{1}{\tau_2} + i\,\omega\right)\left(\frac{1}{\tau_1} + \frac{1}{\tau_2} + i\,\omega\right)}\, S_s(\omega) \tag{72}$$

where $C_s(T) = \langle s(t)\, s(t+T)\rangle$, $C_a(T) = \langle (a(t) - a_0)\,(a(t+T) - a_0)\rangle$, and $C_{as}(T) = \langle (a(t) - a_0)\, s(t+T)\rangle$. The first term in $S_a(\omega)$ comes from responses to signals, the second term comes from filtered particle arrival noise, and the third term comes from internal kinase noise. For convenience, we will define $\tau_3^{-1} = \tau_1^{-1} + \tau_2^{-1}$.

We now need to decompose $S_a(\omega)$ into the product of a causal and an anti-causal part by finding its zeros and poles. The zeros satisfy $S_a(\omega = i\,z_a) = 0$ are complex solutions to the equation:

$$\frac{G_r^2}{\tau_1^2}\left(2\,r_0^2\,g^2\,\frac{\sigma_v^2}{\tau_v} + r_0\,\omega^2\left(\frac{1}{\tau_v^2} + \omega^2\right)\right) + 2\,D_n\left(\frac{1}{\tau_v^2} + \omega^2\right)\left(\frac{1}{\tau_3^2} + \omega^2\right) = 0. \tag{73}$$

This can be written in terms of the particle arrival SNR, $\gamma_r = 2\,r_0\,g^2\,\sigma_v^2\,\tau_v^3$, and the ratio of the diffusivity of filtered particle noise and the diffusivity of slow kinase noise, $R = \frac{1}{2}\frac{G_r^2}{\tau_1^2}\frac{r_0}{D_n}$:

$$R\left(\gamma_r + \tau_v^2\,\omega^2\,(1 + \tau_v^2\,\omega^2)\right) + (1 + \tau_v^2\,\omega^2)\left(\frac{\tau_v^2}{\tau_3^2} + \tau_v^2\,\omega^2\right) = 0 \tag{74}$$

The zeros of $S_a(\omega)$ are:

$$i\,z_{a,1} = i\frac{1}{\tau_v}\frac{1}{\sqrt{2\,(1+R)}}\sqrt{\left(\frac{\tau_v}{\tau_3}\right)^2 + (1+R) - \sqrt{(1+R)\big(1 + R\,(1 - 4\,\gamma_r)\big) - 2\,(1+R)\left(\frac{\tau_v}{\tau_3}\right)^2 + \left(\frac{\tau_v}{\tau_3}\right)^4}},$$

$$i\,z_{a,2} = i\frac{1}{\tau_v}\frac{1}{\sqrt{2\,(1+R)}}\sqrt{\left(\frac{\tau_v}{\tau_3}\right)^2 + (1+R) + \sqrt{(1+R)\big(1 + R\,(1 - 4\,\gamma_r)\big) - 2\,(1+R)\left(\frac{\tau_v}{\tau_3}\right)^2 + \left(\frac{\tau_v}{\tau_3}\right)^4}} \tag{75}$$

as well as their complex conjugates.

The poles of $S_a(\omega)$ satisfy $\frac{1}{S_a(\omega = i\,p_a)} = 0$ and are $i\,p_{a,1} = i\frac{1}{\tau_v}$, $i\,p_{a,2} = i\frac{1}{\tau_2}$, and , $i\,p_{a,3} = i\frac{1}{\tau_3}$, as well as their complex conjugates.

We decompose $S_a(\omega)$ as:

$$S_a(\omega) = \phi_a(\omega)\,\phi_a^*(\omega) \tag{76}$$

where

$$\phi_a(\omega) = \sqrt{2\,D_n\,(1+R)}\frac{\big(z_{a,1} - i\,\omega\big)\big(z_{a,2} - i\,\omega\big)}{\big(p_{a,1} - i\,\omega\big)\,\big(p_{a,2} - i\,\omega\big)\,\big(p_{a,3} - i\,\omega\big)}. \tag{77}$$

Next, we need the causal part of the following (see Appendix A):

$$\frac{S_{as}(\omega)}{\phi_a^*(\omega)} e^{-i\,\omega\,\tau} = -\frac{G_r/\tau_1}{\sqrt{2\,D_n\,(1+R)}} \frac{2\,r_0\,g^2\,\sigma_v^2\,\frac{1}{\tau_v}}{\left(\frac{1}{\tau_v} - i\,\omega\right)\,\left(z_{a,1} + i\,\omega\right)\,\left(z_{a,2} + i\,\omega\right)} e^{-i\,\omega\,\tau} \tag{78}$$

Again, we find the causal part of this expression by doing a partial fraction decomposition and keeping only the terms with poles and zeros that have negative imaginary part:

$$\frac{S_{as}(\omega)}{\phi_a^*(\omega)} e^{-i\,\omega\,\tau} = \frac{A}{\left(\frac{1}{\tau_v} - i\,\omega\right)} + \frac{B}{\left(z_{a,1} + i\,\omega\right)} + \frac{C}{\left(z_{a,2} + i\,\omega\right)}, \tag{79}$$

for unknown A, B, and C. Only the pole of the first term (at $\omega = -i\frac{1}{\tau_v}$) has negative imaginary part, so we only need to compute A to get the causal part of this expression. This is:

$$A = \left[-\frac{G_r/\tau_1}{\sqrt{2\,D_n\,(1+R)}} \frac{2\,r_0\,g^2\,\sigma_v^2\,\frac{1}{\tau_v}}{\left(z_{a,1} + i\,\omega\right)\,\left(z_{a,2} + i\,\omega\right)} e^{-i\,\omega\,\tau}\right]_{\omega=-i\frac{1}{\tau_v}} \tag{80}$$

$$= -\frac{G_r/\tau_1}{\sqrt{2\,D_n\,(1+R)}} \frac{2\,r_0\,g^2\,\sigma_v^2\,\tau_v}{\left(1 + z_{a,1}\,\tau_v\right)\left(1 + z_{a,2}\,\tau_v\right)} e^{-\frac{\tau}{\tau_v}}, \tag{81}$$

and the causal part of $S_{as}(\omega)/\phi_a^*(\omega)$ is then:

$$\left[\frac{S_{as}(\omega)}{\phi_a^*(\omega)} e^{-i\,\omega\,\tau}\right]^+ = -\frac{G_r/\tau_1}{\sqrt{2\,D_n\,(1+R)}} \frac{2\,r_0\,g^2\,\sigma_v^2\,\tau_v}{\left(1 + z_{a,1}\,\tau_v\right)\left(1 + z_{a,2}\,\tau_v\right)} \frac{e^{-\frac{\tau}{\tau_v}}}{\left(\frac{1}{\tau_v} - i\,\omega\right)}. \tag{82}$$

Finally, like $C_{rs}(\tau)$ in the section above, $C_{as}(\tau) \propto \exp\left(-\frac{\tau}{\tau_v}\right)$ when $\tau \geq 0$.

With these expressions, the optimal kernel that computes the mean of $p(s(t+\tau)|\{a\})$ is (Appendix A):

$$M_a(\omega) = \frac{1}{\phi_a(\omega)} \left[\frac{S_{as}(\omega)}{\phi_a^*(\omega)} e^{-i\,\omega\,\tau}\right]^+ \tag{83}$$

$$= -e^{-\frac{\tau}{\tau_v}} \frac{2\frac{G_r}{\tau_1}\,r_0\,g^2\,\sigma_v^2\,\tau_v}{2\,D_n\,(1+R)\,\left(1 + z_{a,1}\,\tau_v\right)\left(1 + z_{a,2}\,\tau_v\right)} \frac{\left(\frac{1}{\tau_2} - i\,\omega\right)\left(\frac{1}{\tau_3} - i\,\omega\right)}{\left(z_{a,1} - i\,\omega\right)\left(z_{a,2} - i\,\omega\right)} \tag{84}$$

We discuss this kernel in the following section.

The variance of $P(s(t+\tau)|\{a\})$, $\sigma_{s|a}^2(\tau)$, is (Appendix A, Eqn. 127):

$$\sigma_{s|a}^2(\tau) = \sigma_s^2 - \frac{1}{2\pi} \int_{-\infty}^{\infty} S_{as}^*(\omega)\,e^{i\,\omega\,\tau}\,M_a(\omega)\,d\omega \tag{85}$$

$$= \sigma_s^2 \left(1 - e^{-2\frac{\tau}{\tau_v}} \frac{2 \frac{G_r^2}{\tau_1^2} r_0^2 \, g^2 \, \sigma_v^2 \, \tau_v^3}{2 \, D_n \, (1+R)\left(1 + z_{a,1} \, \tau_v\right)^2 \left(1 + z_{a,2} \, \tau_v\right)^2} \right), \tag{86}$$

where $\sigma_s^2 = g^2 \, \sigma_v^2$. Therefore, the correlation coefficient $\rho_{as}^2(\tau)$ is:

$$\rho_{as}^2(\tau) = 1 - \frac{\sigma_{s|a}^2(\tau)}{\sigma_s^2} = e^{-2\frac{\tau}{\tau_v}} \frac{2 \frac{G_r^2}{\tau_1^2} r_0^2 \, g^2 \, \sigma_v^2 \, \tau_v^3}{2 \, D_n \, (1+R)\left(1 + z_{a,1} \, \tau_v\right)^2 \left(1 + z_{a,2} \, \tau_v\right)^2}, \tag{87}$$

or in terms of $\gamma_r = 2 \, r_0 \, g^2 \, \sigma_v^2 \, \tau_v^3$ and $R = \frac{1}{2} \frac{G_r^2}{\tau_1^2} \frac{r0}{D_n}$:

$$= e^{-2\frac{\tau}{\tau_v}} \frac{R}{(1+R)} \frac{\gamma_r}{\left(1 + z_{a,1} \, \tau_v\right)^2 \left(1 + z_{a,2} \, \tau_v\right)^2}. \tag{88}$$

Finally, using Eqn. 66 above, we find that the information about current signal encoded in *E. coli*'s kinase activity is:

$$\dot{I}_{s\to a}^* = \frac{1}{\tau_v} \frac{\rho_{as}^2(\tau = 0)}{1 - \rho_{as}^2(\tau = 0)} = \frac{1}{\tau_v} \frac{\frac{R}{(1+R)} \frac{\gamma_r}{\left(1 + z_{a,1} \, \tau_v\right)^2 \left(1 + z_{a,2} \, \tau_v\right)^2}}{1 - \frac{R}{(1+R)} \frac{\gamma_r}{\left(1 + z_{a,1} \, \tau_v\right)^2 \left(1 + z_{a,2} \, \tau_v\right)^2}}. \tag{89}$$

In shallow gradients, $\dot{I}_{s\to a}^* \approx \frac{1}{\tau_v} \rho_{as}^2(\tau = 0) \approx \frac{2}{\tau_v} I\left(\{a(t)\}; s(t)\right)$, and only the leading order g^2 term of $\dot{I}_{s\to a}^*$ contributes to the final expression. Since $\gamma_r \propto g^2$ in Eqn. 88, we can get the shallow-gradient expression for $\dot{I}_{s\to a}^*$ by evaluating $z_{a,1}$ and $z_{a,2}$ at $g = 0$. This is equivalent to taking $\gamma_r \to 0$, which gives:

$$z_{a,1} \approx \frac{1}{\tau_3 \sqrt{1+R}}, \qquad z_{a,2} \approx \frac{1}{\tau_v}. \tag{90}$$

Thus, in shallow gradients, we get:

$$\dot{I}_{s\to a}^* \approx \frac{1}{\tau_v} \frac{1}{4} \frac{R}{1+R} \frac{\gamma_r}{\left(1 + \frac{\tau_v}{\tau_3} \frac{1}{\sqrt{1+R}}\right)^2} = \frac{1}{\tau_v} \frac{1}{4} \frac{\frac{1}{2} \frac{G_r^2}{\tau_1^2} \frac{r_0}{D_n}}{1 + \frac{1}{2} \frac{G_r^2}{\tau_1^2} \frac{r_0}{D_n}} \frac{2 \, r_0 \, g^2 \, \sigma_v^2 \, \tau_v^3}{\left(1 + \frac{\tau_v}{\tau_3}\left(1 + \frac{1}{2} \frac{G_r^2}{\tau_1^2} \frac{r_0}{D_n}\right)^{-1/2}\right)^2}, \tag{91}$$

where again $\tau_3^{-1} = \tau_1^{-1} + \tau_2^{-1}$. Furthermore, since $\tau_1 \ll \tau_v$, taking $\tau_1 \to 0$ only slightly increases the information rate and gives a simpler expression in terms of a kinase signal to noise ratio, $\gamma_a = \frac{G_r^2}{D_n} r_0^2 \, g^2 \, \sigma_v^2 \, \tau_v$, and the particle arrival signal to noise ratio, $\gamma_r = 2 \, r_0 \, g^2 \sigma_v^2 \, \tau_v^3$:

$$\dot{I}^*_{s\to a} \approx \frac{1}{\tau_v}\frac{1}{4}\,\gamma_a \frac{\frac{\gamma_r}{\gamma_a}}{\left(1+\sqrt{\frac{\gamma_r}{\gamma_a}}\right)^2} = \frac{1}{\tau_v}\frac{1}{4}\frac{G_r^2}{D_n} r_0^2\, g^2\, \sigma_v^2\, \tau_v \frac{\frac{2\,D_n\,\tau_v^2}{G_r^2\,r_0}}{\left(1+\sqrt{\frac{2\,D_n\,\tau_v^2}{G_r^2\,r_0}}\right)^2}. \tag{92}$$

We also note that for finite g but $\tau_1 \to 0$, $z_{a,1}$ and $z_{a,2}$ are:

$$z_{a,1} \approx \frac{1}{\tau_v}\frac{1}{\sqrt{2}}\sqrt{1+\frac{\gamma_r}{\gamma_a} - \sqrt{1-4\,\gamma_r - 2\frac{\gamma_r}{\gamma_a} + \left(\frac{\gamma_r}{\gamma_a}\right)^2}},$$

$$z_{a,2} \approx \frac{1}{\tau_v}\frac{1}{\sqrt{2}}\sqrt{1+\frac{\gamma_r}{\gamma_a} + \sqrt{1-4\,\gamma_r - 2\frac{\gamma_r}{\gamma_a} + \left(\frac{\gamma_r}{\gamma_a}\right)^2}}. \tag{93}$$

We plugged these expressions into Eqn. 89, with $\frac{R}{1+R} \to 1$ as $\tau_1 \to 0$, to generate the plots in Fig. 3 of the main text.

Eqns. 44, 52, and 89 for the information rates $\dot{I}^*_{s\to r}$ and $\dot{I}^*_{s\to a}$ are nearly exact, but make several assumptions. They require $r_0\,\tau_v \gg 1$ so that we can approximate particle arrivals as Gaussian. They also use Gaussian approximations for the mutual information quantities $I(s(t);\{r\})$ and $I(s(t);\{a\})$, which are valid when these quantities are small (shallow gradients, small g). We used linear theory to model kinase responses, which is valid if deviations in kinase activity from baseline are small—i.e. when g is small. And we ignored feedbacks in which responses to signals change the signal statistics that the cell experiences, again valid when g is small. Each of these assumptions can break at a different characteristic value of g: for particle arrival rate, small g means $\gamma_r \ll 1$; for kinase activity, small g means $\gamma_a \ll 1$. That all said, Eqns. 44, 52, and 89 currently provide our best analytical insight into information transfer during chemotaxis.

Optimal kernel for estimating signal from kinase activity

To understand the kernel $M_a(\omega)$ that constructs an estimate of the current signal, $s(t)$, from past kinase activity, $\{a\}$, we first multiply it by the kinase response function of particle arrivals, $K_r(\omega)$. This gives a composite kernel that effectively maps the past of particle arrivals r, corrupted by kinase noise, to an estimate of the signal $s(t)$:

$$-M_a(\omega)\,K_r(\omega) = -\frac{2\left(\frac{G_r}{\tau_1}\right)^2 r_0\,g^2\,\sigma_v^2\,\tau_v}{2\,D_n\,(1+R)\,\big(1+z_{a,1}\,\tau_v\big)\big(1+z_{a,2}\,\tau_v\big)}\frac{(-i\,\omega)}{\big(z_{a,1}-i\,\omega\big)\big(z_{a,2}-i\,\omega\big)}. \tag{94}$$

In the time domain, this is:

$$IFT[-M_a(\omega)\,K_r(\omega)] = \frac{2\left(\frac{G_r}{\tau_1}\right)^2 r_0\,g^2\,\sigma_v^2\,\tau_v}{2\,D_n\,(1+R)}\frac{\big(z_{a,2}\,\exp(-z_{a,2}\,t) - z_{a,1}\,\exp(-z_{a,1}\,t)\big)}{\big(1+z_{a,1}\,\tau_v\big)\big(1+z_{a,2}\,\tau_v\big)\big(z_{a,1}-z_{a,2}\big)}\,\Theta(t). \tag{95}$$

This composite kernel that effectively acts on particle arrivals has the same structure as the optimal kernel $M_r(T)$ (Eqn. 53) for directly constructing $s(t)$ from particle arrivals. It's biphasic and adapts perfectly, although with different time scales than $M_r(T)$. This means that $M_a(T)$ attempts to invert the kinase response function $K_r(T)$, to the extent possible given the kinase noise $N_n(T)$, and then apply something as close as possible to the optimal kernel for particle counts, $M_r(T)$.

Taking this line of thinking further, the optimal kernel acting on particle counts, $M_r(T)$, is the kernel that the cell should *try* to implement (up to changes of units). However, the cell has to communicate information about the signal $s(t)$ through multiple chemical species in order to send them from the kinases at one location to the motors at various other locations. These steps impose constraints on the cell's signaling pathway, and they add noise. Despite this, the cell should be attempting to make its composite kernel from input (particle counts) to output (tumble rate) look like $M_r(T)$.

Information about current versus past signals encoded in kinase activity

We previously quantified the information about all past signals encoded in kinase activity, $\dot{I}_{s\to a}$, and found that *E. coli* use this information efficiently: they climb gradients at speeds near the information-performance limit (3). There are two possible inefficiencies that prevent *E. coli* from reaching the limit: first, cells might encode information about past signals, which don't contribute to gradient-climbing; and second, information about current signal can be lost in communication to the motor behavior. Now that we have an expression for the information about current signal $s(t)$ in kinase activity, we can distinguish between these two effects.

We defined the information about all past signals encoded in kinase activity using the following transfer entropy rate:

$$\dot{I}_{s\to a} \equiv \lim_{dt\to 0} \frac{1}{dt} I(a(t+dt); \{s\}|\{a\})\,. \tag{96}$$

The subset of this information that is relevant to chemotaxis is:

$$\dot{I}^{*}_{s\to a} \equiv \lim_{dt\to 0} \frac{1}{dt} I(a(t+dt); s(t)|\{a\})\,, \tag{97}$$

which is the information we have considered here. How do these information rates compare to each other for the kinase response function and noise correlation function that we measured here and previously?

First, note that if kinase activity a were Markovian in $s(t)$, then we would have

$$\begin{aligned} \dot{I}_{s\to a} &= \lim_{dt\to 0} \frac{1}{dt} I(a(t+dt); \{s\}|\{a\}) \\ &= \lim_{dt\to 0} \frac{1}{dt} I(a(t+dt); s(t)|\{a\}) \\ &= \dot{I}^{*}_{s\to a}, \end{aligned} \tag{98}$$

and all information about signals encoded in kinase activity is relevant to gradient climbing. Surprisingly, this means that a long response adaptation time does not necessarily degrade information about the current signal.

We can evaluate both of these information rates for the response and noise models used here. In the regime of shallow gradients and $\tau_2 \approx \tau_n$, the information about past and present signals is (3,25):

$$\dot{I}_{s\to a} \approx \frac{1}{4\pi}\int_{-\infty}^{\infty} \frac{S(\omega)\frac{r_0^2}{\omega^2}|K_r(\omega)|^2}{N_n(\omega) + r_0\,|K_r(\omega)|^2}\,d\omega \tag{99}$$

$$= \frac{\frac{G_r^2}{\tau_1^2}\,r_0^2\,g^2\,\sigma_v^2\,\tau_3^2}{4\,D_n\left(1+\frac{\tau_3}{\tau_v}\sqrt{1+\frac{G_r^2}{\tau_1^2}\frac{r_0}{2\,D_n}}\right)} = \frac{1}{\tau_v}\frac{1}{4}\frac{R\,\gamma_r\left(\frac{\tau_3}{\tau_v}\right)^2}{\left(1+\frac{\tau_3}{\tau_v}\sqrt{1+R}\right)} \tag{100}$$

which we have expressed in terms of the ratio of the diffusivity of filtered particle noise and the diffusivity of slow kinase noise, $R = \frac{G_r^2}{\tau_1^2}\frac{r_0}{2\,D_n}$; the particle arrival signal-to-noise ratio, $\gamma_r = 2\,r_0\,g^2\,\sigma_v^2\,\tau_v^3$; and $\tau_3^{-1} = \tau_1^{-1} + \tau_2^{-1}$.

We compare this to the information about current signal only derived in the previous section, Eqn. 91, reproduced below:

$$\dot{I}^*_{s\to a} = \frac{1}{\tau_v}\frac{1}{4}\frac{R}{1+R}\frac{\gamma_r}{\left(1+\frac{\tau_v}{\tau_3}\frac{1}{\sqrt{1+R}}\right)^2} \tag{101}$$

The ratio of these two information rates has a particularly simple form:

$$\frac{\dot{I}^*_{s\to a}}{\dot{I}_{s\to a}} \approx \frac{\tau_v}{\tau_3\sqrt{1+R}+\tau_v} = \frac{\tau_v}{\tau_3\sqrt{1+\frac{G_r^2}{\tau_1^2}\frac{r_0}{2\,D_n}}+\tau_v}. \tag{102}$$

Thus, for $\dot{I}_{s\to a}$ to mostly carry information about current signal and be close to $\dot{I}^*_{s\to a}$, 1) the time scale of initial kinase response must be short compared to the signal correlation time, $\tau_1 \ll \tau_v$; and 2) the diffusivity of filtered particle noise must be small compared to that of internal kinase noise, $G_r^2\,r_0 \ll 2\,D_n$. Using $\tau_1 = 1/60\,s$ from biochemistry studies Refs. (1,2), we estimate that $\frac{\dot{I}^*_{s\to a}}{\dot{I}_{s\to a}} \approx 0.88 \pm 0.01$ in $c_0 = 1\ \mu\text{M}$, and increases as c_0 gets large or small. This suggests that *E. coli*'s main source of "inefficiency" is that relevant information in kinase activity is lost in communication with the motors.

This result might appear to be in contradiction with the results of Ref. (16), which found that the fraction of predictive information about signals relative to past information about signals was very small (about 1%) in a model of *E. coli*'s kinase activity, a, and downstream readout molecules, x (CheYp). (Our $\dot{I}^*_{s\to a}$, being a predictive information rate, is very similar to their predictive information, while $\dot{I}_{s\to a}$ is very similar to their past information.) However, that study considered predictive and past information encoded in

the *current* value of the readout molecule, $x(t)$, instead of the entire history of readout molecules $\{x\}$. This difference in how our information quantities are defined explains the large difference.

Kinase activity a and even CheY phosphorylation level downstream x are not the final outputs of the chemotaxis system. Instead, downstream pathway dynamics can act on the entire past of a or x to extract more information and make behavioral decisions. Therefore, the current values of $a(t)$ and $x(t)$ do not need to be faithful estimates of the current (or future) signal $s(t)$; they just need to carry decodable information about $s(t)$ in their trajectories. Our information measures above account for this.

In summary, we have two sets of inequalities. The first set of inequalities,

$$\dot{I}_{s\to a} \geq \dot{I}^*_{s\to a} \geq \dot{I}^*_{s\to m} \propto \left(\frac{v_d}{v_0}\right)^2, \tag{103}$$

was the focus of our previous work (3), and it quantifies how efficiently *E. coli* use the information *that they have* at the level of kinase activity, $\dot{I}_{s\to a}$, to climb gradients. The main result of that work was that $\dot{I}_{s\to a} \approx 2\,\dot{I}^*_{s\to m}$. The analysis above adds to this: $\dot{I}_{s\to a} \approx \dot{I}^*_{s\to a} \approx 2\,\dot{I}^*_{s\to m}$.

The second set of inequalities,

$$\dot{I}^*_{s\to r} \geq \dot{I}^*_{s\to a} \geq \dot{I}^*_{s\to m} \propto \left(\frac{v_d}{v_0}\right)^2, \tag{104}$$

particularly the left-most one, is the focus of this work. It quantifies how much information *E. coli* get compared to the physical limit. The main result of this manuscript is that $\dot{I}^*_{s\to r} \gg \dot{I}^*_{s\to a}$.

Appendix A: Causal Wiener filter derivation

Causal Wiener filtering theory seeks a linear estimator of an unknown quantity $s(t+\tau)$ at time τ in the future, from past observations of a quantity x that is correlated with s (26). The past of x is denoted $\{x(t)\}$. Both s and x are assumed to be stationary stochastic processes with zero means: $\langle x(t)\rangle = \langle s(t)\rangle = 0$. The Wiener filter, $M_x(T)$, is the kernel that minimizes the mean squared error of the estimator:

$$M_x(T) = \underset{K(T)}{\operatorname{argmin}}\ \langle e^2(\tau)\rangle = \underset{K(T)}{\operatorname{argmin}} \left\langle \left(s(t+\tau) - \int_{-\infty}^{t} K(t-t')\,x(t')\,dt' \right)^2 \right\rangle. \tag{105}$$

In general, the estimator of $s(t+\tau)$ that minimizes the mean squared error is the conditional mean $\langle s(t+\tau)|\{x(t)\}\rangle$. In the case of Gaussian-distributed s and x, the conditional mean $\langle s(t+\tau)|\{x(t)\}\rangle$ is exactly a linear function of $\{x(t)\}$, so the linear estimator above is the global optimum. The minimum error $\langle e^*(\tau)^2\rangle = \sigma^2_{s|x}(\tau)$ is the conditional variance of $s(t+\tau)$ given past x. The main technical challenge of finding the optimal kernel is the constraint that it must be causal: $M_x(T) = 0$ for $T < 0$.

To derive the optimal kernel, first we expand the square in the objective function:

$$\langle e^2(\tau)\rangle = \left\langle s(t+\tau)^2 - 2\,s(t+\tau)\int_{-\infty}^{\infty} K(t-t')\,x(t')\,dt' + \int_{-\infty}^{\infty} K(t-t')\,x(t')\,dt' \int_{-\infty}^{\infty} K(t-t'')\,x(t'')\,dt'' \right\rangle, \tag{106}$$

and move the expectation inside of the integrals:

$$= \sigma_s^2 - 2\int_{-\infty}^{\infty} K(t-t')\,\langle s(t+\tau)\,x(t')\rangle\,dt' + \int_{-\infty}^{\infty}\int_{-\infty}^{\infty} K(t-t')K(t-t'')\,\langle x(t')\,x(t'')\rangle\,dt'\,dt''\,. \quad (107)$$

Here we used time-translation invariance of s: $\langle s(t+\tau)^2\rangle = \langle s(t)^2\rangle = \sigma_s^2$. Next, change variables to $t' \to \tau' = t - t'$ and $t'' \to \tau'' = t - t''$, replacing absolute time with time delays. τ' and $\tau'' > 0$ correspond to time delay into the past.

$$= \sigma_s^2 - 2\int_{-\infty}^{\infty} K(\tau')\,\langle s(t+\tau)\,x(t-\tau')\rangle\,d\tau' + \int_{-\infty}^{\infty}\int_{-\infty}^{\infty} K(\tau')K(\tau'')\,\langle x(t-\tau')\,x(t-\tau'')\rangle\,d\tau'\,d\tau''\,. \quad (108)$$

Defining the cross-correlation function $C_{xs}(t-t') = \langle x(t')\,s(t)\rangle$ and autocorrelation function $C_x(t-t') = \langle x(t')\,x(t)\rangle$:

$$= \sigma_s^2 - 2\int_{-\infty}^{\infty} K(\tau')\,C_{xs}(\tau'+\tau)\,d\tau' + \int_{-\infty}^{\infty}\int_{-\infty}^{\infty} K(\tau')K(\tau'')\,C_x(\tau''-\tau')\,d\tau'\,d\tau''\,. \quad (109)$$

When $\tau > 0$ in $C_{xy}(\tau)$, y is evaluated at a time point in the future relative to x. Note that $C_{sx}(\tau) = C_{xs}(-\tau)$.

Next, we take the functional derivative of the mean squared error with respect to $K(T)$:

$$\frac{\delta\langle e^2(\tau)\rangle}{\delta K} = -2\,C_{xs}(\tau'+\tau) + \int_{-\infty}^{\infty} K(\tau'')\,C_x(\tau''-\tau')\,d\tau'' + \int_{-\infty}^{\infty} K(\tau'')\,C_x(\tau'-\tau'')\,d\tau'' \quad (110)$$

Since $C_x(\tau) = C_x(-\tau)$, this is:

$$= -2\,C_{xs}(\tau'+\tau) + 2\int_{-\infty}^{\infty} K(\tau'')\,C_x(\tau''-\tau')\,d\tau''\,. \quad (111)$$

Now we need to consider the causal constraint on $K(T)$. For optimality with this constraint, the equation above must equal zero for $\tau' \geq 0$ (at times when x precedes s in C_{xs}). Otherwise, for $\tau' < 0$, the derivative is not necessarily zero. Therefore, at the optimum we can write (27):

$$\int_{-\infty}^{\infty} M_x(\tau'')\,C_x(\tau'-\tau'')\,d\tau'' - C_{xs}(\tau'+\tau) = A(\tau') \quad (112)$$

where

$$A(\tau') = \begin{cases} 0, & \tau' \geq 0 \\ a(\tau'), & \tau' < 0 \end{cases} \quad (113)$$

and $a(\tau')$ is some unspecified function. $A(\tau')$ is therefore anti-causal – it is only non-zero at times in the future ($\tau' < 0$). At first glance, this optimality condition might seem less constrained than if $A(\tau')$ were zero for all τ' (the optimality condition for the optimal non-causal filter). However, the fact that $A(\tau')$ is nonzero for $\tau' < 0$ actually limits the space of filters $M_x(\tau)$ that keep $A(\tau') = 0$ for $\tau' \geq 0$, as we will see below.

Next, we take the Fourier transform of both sides, defined as $f(\omega) = F[f(t)] = \int_{-\infty}^{\infty} f(t)\,e^{i\,\omega\,t}\,dt$. Convolutions in the time domain become element-wise products in the Fourier domain:

$$M_x(\omega)\, C_x(\omega) = C_{xs}(\omega)\, e^{-i\,\omega\,\tau} + A(\omega). \tag{114}$$

On the left-hand side, we have the product of a causal function and a function that is nonzero for positive and negative time delays, the result of which is also nonzero for positive and negative time delays. On the right-hand side, we have a function that is nonzero for positive and negative time delays and an anti-causal function. How do we get the optimal causal kernel $M_x(\omega)$ out of this?

Naively, one might divide both sides by $C_x(\omega)$ and then multiply element-wise by a Heaviside step function in the time domain to get a causal kernel $M_x(\omega)$. However, although the resulting kernel is causal, it does not satisfy the optimality condition. Plugging that kernel back into Eqn. 114, it multiplies the non-causal $C_x(\omega)$, and the result is non-causal. Thus, $A(\omega)$ is non-causal, so that kernel does not satisfy the optimality condition, $A(\tau') = 0$ for $\tau' \geq 0$.

Instead, we need to split $C_x(\omega)$ into causal and anti-causal parts, called a spectral factorization or Wiener-Hopf factorization (17–19):

$$C_x(\omega) = \phi(\omega)\, \phi^*(\omega), \tag{115}$$

where $\phi(\omega)$ is a causal function in the time domain and its complex conjugate $\phi^*(\omega)$ is anti-causal. $\phi(\omega)$ is constructed by putting all poles and zeros of $C_x(\omega)$ with negative real part into $\phi(\omega)$ and those with positive real part into $\phi^*(\omega)$.

Plugging this into the optimality condition:

$$M_x(\omega)\, \phi(\omega)\, \phi^*(\omega) = C_{xs}(\omega)\, e^{-i\,\omega\,\tau} + A(\omega) \tag{116}$$

$$M_x(\omega)\, \phi(\omega) = \frac{C_{xs}(\omega)}{\phi^*(\omega)}\, e^{-i\,\omega\,\tau} + \frac{A(\omega)}{\phi^*(\omega)}. \tag{117}$$

The left-hand side is now a causal function in the time domain, being the product of causal functions, and the right-hand side contains a non-causal function and an anti-causal function.

Multiplying both sides of Eqn. 117 by a Heaviside function in the time domain and then transforming back to Fourier space eliminates the anti-causal term $\frac{A(\omega)}{\phi^*(\omega)}$ and leaves the left-hand side unaffected:

$$M_x(\omega)\, \phi(\omega) = \left[\frac{C_{xs}(\omega)}{\phi^*(\omega)} e^{-i\,\omega\,\tau}\right]^+. \tag{118}$$

Now the right-hand side is causal, and dividing by $\phi(\omega)$ gives the optimal causal filter:

$$M_x(\omega) = \frac{1}{\phi(\omega)} \left[\frac{C_{xs}(\omega)}{\phi^*(\omega)} e^{-i\,\omega\,\tau}\right]^+. \tag{119}$$

To check that this filter satisfies the optimality condition (Eqn. 114), we can plug it in:

$$\frac{1}{\phi(\omega)} \left[\frac{C_{xs}(\omega)}{\phi^*(\omega)} e^{-i\,\omega\,\tau}\right]^+ \phi(\omega)\, \phi^*(\omega) = C_{xs}(\omega)\, e^{-i\,\omega\,\tau} + A(\omega) \tag{120}$$

$$\left[\frac{C_{xs}(\omega)}{\phi^*(\omega)} e^{-i\,\omega\,\tau}\right]^+ \phi^*(\omega) = C_{xs}(\omega)\, e^{-i\,\omega\,\tau} + A(\omega) \tag{121}$$

$$\left[\frac{C_{xs}(\omega)}{\phi^*(\omega)} e^{-i\,\omega\,\tau}\right]^+ = \frac{C_{xs}(\omega)}{\phi^*(\omega)}\, e^{-i\,\omega\,\tau} + \frac{A(\omega)}{\phi^*(\omega)}. \tag{122}$$

Now the left-hand side is the causal part of the first term on the right-hand side. Therefore, their difference is anti-causal and $A(\omega)$ is thus anti-causal, as desired:

$$A(\omega) = -\phi^*(\omega)\left[\frac{C_{xs}(\omega)}{\phi^*(\omega)} e^{-i\,\omega\,\tau}\right]^- . \tag{123}$$

At the optimum, the mean square error $\langle e^2(\tau)\rangle = \sigma_{s|x}^2(\tau)$ is:

$$\sigma_{s|x}^2(\tau) = \sigma_s^2 - 2\int_0^\infty M_x(\tau')\, C_{xs}(\tau' + \tau)\, d\tau' + \int_0^\infty \int_0^\infty M_x(\tau')\, M_x(\tau'')\, C_x(\tau'' - \tau')\, d\tau'\, d\tau'' , \tag{124}$$

where we have set the lower limit to zero because the kernel $M_x(T)$ is zero for $T < 0$. Using the optimality condition $\int_0^\infty M_x(\tau'')\, C_x(\tau'' - \tau')\, d\tau'' - C_{xs}(\tau' + \tau) = 0$ for $\tau' \geq 0$, we get:

$$= \sigma_s^2 - \int_0^\infty M_x(\tau')\, C_{xs}(\tau' + \tau)\, d\tau' . \tag{125}$$

Since $C_{xs}(\tau) = C_{sx}(-\tau)$, this is:

$$= \sigma_s^2 - \int_{-\infty}^\infty M_x(\tau')\, C_{sx}(-\tau - \tau')\, d\tau' , \tag{126}$$

which is the convolution of $M_x(T)$ and $C_{sx}(T)$, with the result evaluated at $-\tau$. This can be expressed using their Fourier transforms (note the minus sign in front of tau in equation (112) leads to a plus sign in the exponent below) as:

$$\sigma_{s|x}^2(\tau) = \sigma_s^2 - \frac{1}{2\pi}\int_{-\infty}^\infty M_x(\omega)\, C_{sx}(\omega)\, e^{i\,\omega\,\tau}\, d\omega . \tag{127}$$

Plugging in the optimal kernel:

$$= \sigma_s^2 - \frac{1}{2\pi}\int_{-\infty}^\infty \frac{C_{sx}(\omega)}{\phi(\omega)}\left[\frac{C_{xs}(\omega)}{\phi^*(\omega)} e^{-i\,\omega\,\tau}\right]^+ e^{i\,\omega\,\tau}\, d\omega . \tag{128}$$

Finally, the correlation coefficient is:

$$\rho_{xs}^2(\tau) = 1 - \frac{\sigma_{s|x}^2(\tau)}{\sigma_s^2} = \frac{1}{\sigma_s^2}\frac{1}{2\pi}\int_{-\infty}^\infty \frac{C_{sx}(\omega)}{\phi(\omega)}\left[\frac{C_{xs}(\omega)}{\phi^*(\omega)} e^{-i\,\omega\,\tau}\right]^+ e^{i\,\omega\,\tau}\, d\omega . \tag{129}$$

Supplemental References

1. Levit MN, Grebe TW, Stock JB. Organization of the Receptor-Kinase Signaling Array That Regulates Escherichia coli Chemotaxis. J Biol Chem. 2002 Sep 27;277(39):36748–54.

2. Francis NR, Levit MN, Shaikh TR, Melanson LA, Stock JB, DeRosier DJ. Subunit Organization in a Soluble Complex of Tar, CheW, and CheA by Electron Microscopy. J Biol Chem. 2002 Sep 27;277(39):36755–9.

3. Mattingly HH, Kamino K, Machta BB, Emonet T. Escherichia coli chemotaxis is information limited. Nat Phys. 2021 Dec;17(12):1426–31.

4. Cover TM, Thomas JA. Elements of Information Theory. New York, NY: Wiley-Interscience; 1991.

5. Sigtermans D. Towards a Framework for Observational Causality from Time Series: When Shannon Meets Turing. Entropy. 2020 Apr;22(4):426.

6. Bialek W, De Ruyter Van Steveninck RR, Tishby N. Efficient representation as a design principle for neural coding and computation. In: 2006 IEEE International Symposium on Information Theory. 2006. p. 659–63.

7. Palmer SE, Marre O, Berry MJ, Bialek W. Predictive information in a sensory population. PNAS. 2015 Jun 2;112(22):6908–13.

8. Becker NB, Mugler A, ten Wolde PR. Optimal Prediction by Cellular Signaling Networks. Phys Rev Lett. 2015 Dec 17;115(25):258103.

9. Cardoso JF. Dependence, Correlation and Gaussianity in Independent Component Analysis. J Mach Learn Res. 2003;4:1177–203.

10. Carlet C, Danger JL, Guilley S, Maghrebi H, Prouff E. Achieving side-channel high-order correlation immunity with leakage squeezing. J Cryptogr Eng. 2014 Jun 1;4(2):107–21.

11. Rioul O, Cheng W, Guilley S. Cumulant Expansion of Mutual Information for Quantifying Leakage of a Protected Secret. In: 2021 IEEE International Symposium on Information Theory (ISIT). 2021. p. 2596–601.

12. Berg HC, Purcell EM. Physics of chemoreception. Biophysical Journal. 1977 Nov 1;20(2):193–219.

13. ten Wolde PR, Becker NB, Ouldridge TE, Mugler A. Fundamental Limits to Cellular Sensing. J Stat Phys. 2016 Mar 1;162(5):1395–424.

14. Mora T, Nemenman I. Physical Limit to Concentration Sensing in a Changing Environment. Phys Rev Lett. 2019 Nov 5;123(19):198101.

15. Hinczewski M, Thirumalai D. Cellular Signaling Networks Function as Generalized Wiener-Kolmogorov Filters to Suppress Noise. Phys Rev X. 2014 Oct 29;4(4):041017.

16. Tjalma AJ, Galstyan V, Goedhart J, Slim L, Becker NB, ten Wolde PR. Trade-offs between cost and information in cellular prediction. Proceedings of the National Academy of Sciences. 2023 Oct 10;120(41):e2303078120.

17. Wiener N, Masani P. The prediction theory of multivariate stochastic processes: I. The regularity condition. Acta Mathematica. 1957 Jan;98(none):111–50.

18. Bialek W. Biophysics: Searching for Principles [Internet]. Princeton University Press; 2012 [cited 2020 Jun 18]. Available from: https://press.princeton.edu/books/hardcover/9780691138916/biophysics

19. Oppenheim A, Verghese G. Signals, Systems and Inference. 1st ed. Pearson; 2016. (Prentice Hall Signal Processing Series).

20. Kalman RE. A New Approach to Linear Filtering and Prediction Problems. Journal of Basic Engineering. 1960 Mar 1;82(1):35–45.

21. Kalman RE, Bucy RS. New Results in Linear Filtering and Prediction Theory. Journal of Basic Engineering. 1961 Mar 1;83(1):95–108.

22. Keegstra JM, Kamino K, Anquez F, Lazova MD, Emonet T, Shimizu TS. Phenotypic diversity and temporal variability in a bacterial signaling network revealed by single-cell FRET. Barkai N, editor. eLife. 2017 Dec 12;6:e27455.

23. Colin R, Rosazza C, Vaknin A, Sourjik V. Multiple sources of slow activity fluctuations in a bacterial chemosensory network. Barkai N, editor. eLife. 2017 Dec 12;6:e26796.

24. Yuan J, Branch RW, Hosu BG, Berg HC. Adaptation at the output of the chemotaxis signalling pathway. Nature. 2012 Apr;484(7393):233–6.

25. Tostevin F, ten Wolde PR. Mutual Information between Input and Output Trajectories of Biochemical Networks. Phys Rev Lett. 2009 May 27;102(21):218101.

26. Wiener N. Extrapolation, Interpolation, and Smoothing of Stationary Time Series: With Engineering Applications. Cambridge, MA, USA: MIT Press; 1949. 163 p.

27. Havlicek JP. Module 4: The Wiener Filter [Internet]. Kalman Filtering. 2005. Available from: https://coecs.ou.edu/Joseph.P.Havlicek/Kalman/notes/KalmanModule4.pdf

96. Mattingly HH, Emonet T. Collective behavior and nongenetic inheritance allow bacterial populations to adapt to changing environments. Proceedings of the National Academy of Sciences. 2022 Jun 28;119(26):e2117377119.

97. Armstrong JB, Adler J, Dahl MM. Nonchemotactic Mutants of Escherichia coli. Journal of Bacteriology. 1967;93(1):390–8.

98. Bachmann BJ. Pedigrees of Some Mutant Strains of Escherichia coli K-12. 1972;36:33.

99. Barker CS, Prüß BM, Matsumura P. Increased Motility of Escherichia coli by Insertion Sequence Element Integration into the Regulatory Region of the flhD Operon. Journal of Bacteriology. 2004 Nov 15;186(22):7529–37.

100. Briegel A, Ortega DR, Tocheva EI, Wuichet K, Li Z, Chen S, et al. Universal architecture of bacterial chemoreceptor arrays. Proceedings of the National Academy of Sciences. 2009 Oct 6;106(40):17181–6.

101. Waite AJ, Frankel NW, Emonet T. Behavioral Variability and Phenotypic Diversity in Bacterial Chemotaxis. Annual Review of Biophysics. 2018;47(1):595–616.

102. Li L, Zhang X, Sun Y, Ouyang Q, Tu Y, Luo C. Phenotypic Variability Shapes Bacterial Responses to Opposing Gradients. PRX Life. 2024 Jan 9;2(1):013001.

103. Qin D, Xia Y, Whitesides GM. Soft lithography for micro- and nanoscale patterning. Nature Protocols. 2010 Mar;5(3):491–502.

104. Edelstein A, Amodaj N, Hoover K, Vale R, Stuurman N. Computer Control of Microscopes Using µManager. Current Protocols in Molecular Biology. 2010;92(1):14.20.1-14.20.17.

105. Zal T, Gascoigne NRJ. Photobleaching-Corrected FRET Efficiency Imaging of Live Cells. Biophysical Journal. 2004 Jun 1;86(6):3923–39.
